# Physical training reduces cell senescence and associated insulin resistance in skeletal muscle

**DOI:** 10.1016/j.molmet.2025.102130

**Published:** 2025-03-22

**Authors:** Agnieszka Podraza-Farhanieh, Rosa Spinelli, Federica Zatterale, Annika Nerstedt, Silvia Gogg, Matthias Blüher, Ulf Smith

**Affiliations:** 1 Lundberg Laboratory for Diabetes Research, Department of Molecular and Clinical Medicine, Sahlgrenska Academy, University of Gothenburg, Gothenburg, 41345, Sweden; 2 Department of Translational Medical Sciences, Federico II University of Naples, Naples, 80131, Italy; 3 Helmholtz Institute for Metabolic, Obesity and Vascular Research of the Helmholtz Zentrum München at the University of Leipzig and University Hospital Leipzig, Leipzig, 04103, Germany

**Keywords:** Cellular senescence, Skeletal muscles, Satellite cells, Obesity, Aging, Exercise intervention

## Abstract

**Background:**

Cell senescence (CS) is a key aging process that leads to irreversible cell cycle arrest and an altered secretory phenotype. In skeletal muscle (SkM), the accumulation of senescent cells contributes to sarcopenia. Despite exercise being a known intervention for maintaining SkM function and metabolic health, its effects on CS remain poorly understood.

**Objectives:**

This study aimed to investigate the impact of exercise on CS in human SkM by analyzing muscle biopsies from young, normal-weight individuals and middle-aged individuals with obesity, both before and after exercise intervention.

**Methods:**

Muscle biopsies were collected from both groups before and after an exercise intervention. CS markers, insulin sensitivity (measured with euglycemic clamp), and satellite cell markers were analyzed. Additionally, *in vitro* experiments were conducted to evaluate the effects of cellular senescence on human satellite cells, focusing on key regulatory genes and insulin signaling.

**Results:**

Individuals with obesity showed significantly elevated CS markers, along with reduced expression of *GLUT4* and *PAX7*, indicating impaired insulin action and regenerative potential. Exercise improved insulin sensitivity, reduced CS markers, and activated satellite cell response in both groups. In vitro experiments revealed that senescence downregulated key regulatory genes in satellite cells and impaired insulin signaling by reducing the Insulin Receptor β-subunit.

**Conclusions:**

These findings highlight the role of CS in regulating insulin sensitivity in SkM and underscore the therapeutic potential of exercise in mitigating age- and obesity-related muscle dysfunction. Targeting CS through exercise or senolytic agents could offer a promising strategy for improving metabolic health and combating sarcopenia, particularly in at-risk populations.

## Highlights

• Obesity, like aging, increases cell senescence in skeletal muscle.
• Muscle senescence links to insulin resistance and metabolic dysfunction.
• Senescence impairs satellite cells, impacting their insulin sensitivity.
• Exercise reduces senescence-related markers, enhancing muscle function and insulin sensitivity.
• Exercise lowers senescence-related markers, supporting overall physical fitness.

## 1 Introduction

Cell senescence (CS) is a conserved aging mechanism characterized by the irreversible arrest of the cell cycle along with alterations in cell function and the secretion of pro-inflammatory factors collectively known as the senescence-associated secretory phenotype (SASP) [[1], [2], [3]]. This process contributes to chronic inflammation, tissue dysfunction and a reduced capacity for cell regeneration [2,4,5]. As individuals age, senescent cells accumulate in various tissues, including skeletal muscle (SkM), impairing muscle function and leading to sarcopenia, the age-related loss of muscle mass and strength [[6], [7], [8]]. Impairment of SkM function can lead to significant metabolic disturbances [[9], [10], [11]]. Since SkM is a primary site for glucose uptake, dysfunction in this tissue results in reduced insulin responsiveness, contributing to metabolic disorders such as type 2 diabetes (T2D) [12,13]. This highlights the importance of maintaining muscle health to prevent adverse metabolic outcomes.

Obesity is a well-established risk factor for numerous chronic diseases which can accelerate the onset of aging in several metabolic tissues, including SkM, by promoting CS [[14], [15], [16]]. Indeed, obesity triggers local tissue inflammation, oxidative stress and metabolic abnormalities, which are key drivers of CS [17] also in SkM [18]. Chronic low-grade inflammation originating from the adipose tissue in obesity, as well as insulin resistance and altered muscle metabolism, are factors that can contribute to the acceleration of muscle aging and dysfunction [19].

CS can impact multiple cell types within SkM, including muscle stem cells (satellite cells), fibro-adipogenic progenitors and resident immune cells, each of which plays a crucial role in muscle regeneration and maintenance [8,14]. Satellite cells, which are normally quiescent, become activated in response to muscle injury or stress leading to proliferation and differentiation into new muscle fibers, thereby playing a critical role in skeletal muscle generation and repair [20]. Thus, senescence in satellite cells can have profound consequences on SkM health, leading to diminished muscle maintenance, impaired regeneration, reduced responsiveness to exercise and increased metabolic dysfunction [8,16].

Regular physical exercise is a highly effective strategy for preserving SkM function and metabolic health, while also reducing several chronic diseases associated with age [21,22]. Exercise interventions have also been shown to reduce circulating biomarkers of CS in man [23] and the burden of senescent cells linked to aging and age-related conditions in colon mucosa [24]. However, very little is known about the impact of exercise on CS in SkM itself. Understanding if exercise may influence senescence markers in SkM is crucial, as it could provide insights into mechanisms that promote healthy aging of SkM and improve metabolic health.

In this study, we investigated the effects of physical exercise on CS markers in human SkM by analyzing muscle biopsies from people with normal body weight and with obesity, before and after regular exercise. Notably, physical intervention led to significant improvements in metabolic parameters, a reduction in CS markers and activation of satellite cell responses. Moreover, *in vitro* experiments demonstrated that senescence negatively impacts satellite cells by reducing key regulatory genes and impairing insulin signaling. Together these findings underscore the critical role of CS in regulating insulin sensitivity and highlight the potential of physical exercise as a therapeutic strategy to mitigate these effects in human.

## 2 Materials and methods

### 2.1 Human participants

We examined a cohort of 55 Caucasian men and women with either normal weight (n = 23) or obesity (n = 32), but without diabetes, who also did not have a history of acute or chronic inflammatory diseases, alcohol or drug abuse. All initial blood samples and SkM biopsies were collected in the morning, between 08:00 and 10:00, following an overnight fast. SkM samples were obtained under local anesthesia from the right vastus lateralis muscle and were promptly frozen in liquid nitrogen as described previously [25].

### 2.2 Exercise interventions

The subgroup of lean individuals (n = 23, all men) was subjected to supervised physical training, which consisted of training sessions on 5 consecutive days of the week for 4 weeks, i.e., 20 sessions in total. Each session included 20 min of biking or running, 45 min of circuit training, and 20 min periods for warming up and cooling down. All subjects completed a graded bicycle test to volitional exhaustion and had maximal oxygen uptake measured with an automated open circuit gas analysis system at baseline. The highest oxygen uptake per minute reached was defined as the maximal oxygen uptake (VO2 maximum), and subjects subsequently trained at their individual submaximal heart rate using heart rate monitors. An additional subgroup of participants with obesity (n = 32; 20 men and 12 women) underwent a structured supervised mixed strengths and endurance training for six months. For warm-up, participants completed 10 min of either bicycle ergometer, stepper machine, rowing device or treadmill at ∼50% of the maximal heart rate determined by the baseline incremental exercise test on a treadmill for time to exhaustion and in the ergospirometry. For cool-down, participants performed 10–15 min of low intensity stretching exercises. The supervised endurance training consisted of 6 exercises á 10 min on treadmill, stepper, cross-trainer, rowing device or bicycle ergometer training. The interval between exercise bouts was 1–5 min. Switching between different training machines was allowed. Heart rate during the exercise was used to prescribe, adjust and control the endurance training. Training intensity recommendations were guided by the individual performance, and training intensity was adjusted to the heart rate. The goal was to achieve a training intensity of ∼75% of maximal intensity. Protocols were adjusted every 4 weeks or at the discretion of the supervisor based on the individual performance of the study participant. For strength training, 10 strength training machines and 5 exercise on dumbbells or therapeutic bands were selected to target the major muscle groups: chest, back, legs, shoulders, biceps, triceps, and the trunk. Strength training consisted of supervised, circle training for 40–60 min, twice a week. Training sessions were monitored and participants received recommendations how to perform exercises. Per exercise, the number of series was 8–12 with 3 repetitions. The interval between series was 30 s and between exercises 1 min. Monitoring was achieved by supervision of each training session. Before the training period, participants performed a 10-repetition maximum test to define the target intensity of ∼75–85% for each exercise. The intensity for each exercise was adjusted every week according to the individual performance based on the subjectively reported residual power after the 10th repetition of the exercise. General training protocols, i.e. the type of exercise, number of series and repetitions were not changed during the 6 months, whereas intensities were adjusted according to the individual performance.

Study participants were instructed to avoid vigorous exercise 48 h prior to blood drawing and muscle biopsy collection. Before entering the exercise program, incremental cycle ergometer tests were performed until exhaustion to assess the maximal power output (Pmax) and ergospirometry (Aeroman professional, Aerolution; Berlin, Germany) to define aerobic capacity and subsequent individual training intensities. In brief, the test started with a 0 W load, which increased every second by another ∼0.278 W (corresponding to 50 W·3 min−1). The minimum cadence was set at 60 rpm. The effort continued until volitional exhaustion. The maximal workload (Wmax) was calculated as the multiplication of the test duration (s) and the load-increasing coefficient. The total work performed was calculated based on the obtained maximal power and the test duration [26].

In addition, we asked the study participants before taking part in the study about their regular physical activity. Because there was no relevant difference in the daily physical activity (e.g., mainly sitting activities at work) or regular exercise level (e.g., <120 min dedicated exercise sessions per week) between individuals in both the lean participants and those in the 6 months obesity program, we did not have to stratify the study participants by baseline physical activity level differences.

### 2.3 Sample collection and metabolic assessments

Fasting blood and SkM samples were obtained at both the start of the study and 48 h following the training period. Blood samples were obtained after an overnight fast, with subjects in a supine position for 30 min, to measure fasting plasma insulin (FPI), hemoglobin A1c (HbA1c), adiponectin, leptin, free fatty acids (FFA), total cholesterol (TC), high-density lipoprotein cholesterol (HDL-C), low-density lipoprotein cholesterol (LDL-C), triglycerides (TG), and high-sensitivity C-reactive protein (hsCRP) using previously described methods [27]. Insulin sensitivity was assessed in all subjects at baseline and after the exercise period using the euglycemic-hyperinsulinemic clamp method, as previously described [28]. Clamps were performed 3 days after the last exercise session. 2-hour oral glucose tolerance tests (OGTT) were performed at baseline and after the exercise program after an overnight fast with 75g standardized glucose solution (Accu Chek Dextrose OGT, Roche, Mannheim, Germany). Percentage body fat and fat free mass were assessed by bioimpedance analysis. Incremental cycle ergometer tests were performed until exhaustion to assess the maximal power output (Pmax), VO2max and ergospirometry (Aeroman professional, Aerolution; Berlin, Germany) to define aerobic capacity and subsequent individual training intensities.

### 2.4 Cell culture

#### 2.4.1 Human skeletal muscle satellite cells (HSkMSC)

HSkMSC, a human primary cell line purchased from Innoprot, were cultured in Skeletal Muscle Cell Medium supplemented with 5% Fetal Bovine Serum (FBS), 1% Penicillin-Streptomycin, and 1% Skeletal Muscle Cell Growth Supplement. Cells were grown at 37 °C in a humidified atmosphere with 5% CO_2_ in T75 flasks or 12-well plates. For experimental consistency, HSkMSC were used from passage 2 to passage 6.

#### 2.4.2 Murine myoblast (C2C12) cells

C2C12 cells were purchased from ATCC and cultured in Dulbecco's Modified Eagle Medium (DMEM) (high glucose) supplemented with 10% Fetal Bovine Serum (FBS) and 1% Penicillin-Streptomycin, as described previously [29]. Cells were grown at 37 °C in a humidified atmosphere with 5% CO_2_ in T75 flasks or 12-well plates. For experimental consistency, C2C12 cells were used at passages below 10. Cells were passaged when they reached 70–80% confluency to avoid overcrowding.

#### 2.4.3 Differentiation of myoblasts

C2C12 cells were cultured in DMEM until they reached 70–80% confluency. At this point, the medium was replaced with differentiation medium (DMEM supplemented with 2% FBS and 1% Penicillin-Streptomycin), as described previously [29]. The medium was changed every 48 h to maintain optimal conditions for differentiation.

#### 2.4.4 Induction of CS with doxorubicin

HSkMSC and C2C12 cells were treated with different concentrations of doxorubicin (DOX) (Sigma–Aldrich) for 2 h in the appropriate media, as previously described [30]. After treatment, the cells were washed once with PBS and replenished with fresh media, followed by incubation for either 24 or 48 h. Conditioned medium (CM) was collected 24h or 48h after exposure to DOX as described. CM from both control and DOX-treated cells were collected. Cell debris was removed by centrifugation at 1200 rpm for 3 min, and the resulting media were stored at −80 °C until further use. To treat naïve HSkMSC, CM was mixed with complete Skeletal Muscle Cell Medium at a ratio of 1:1 and used for 24h or 48h.

#### 2.4.5 Senolytic treatment

Dasatinib (D) (Sigma–Aldrich) was dissolved in DMSO to prepare a 50 mM stock solution, and Quercetin (Q) (Sigma–Aldrich) was similarly dissolved in DMSO, as previously described [31]. HSkMSC were treated with 0.5 μM DOX for 2 h in the appropriate media. After treatment, the cells were washed once with PBS and replenished with fresh media, followed by a 24-hour incubation. At this time point, Dasatinib (200 nM) and Quercetin (50 μM) were added to the media, while fresh media without DQ was added to the remaining cells, followed by an additional 24-hour incubation. At the indicated time points, cells were collected.

#### 2.4.6 Salbutamol treatment

Salbutamol (Sigma–Aldrich; S8260) was dissolved in methanol to prepare a 100 mM stock solution and used directly on the cells. HSkMSC were treated with Salbutamol at a concentration of 50 μM for 2 h in the appropriate media. At the indicated time points, cells were collected.

#### 2.4.7 Insulin/IGF signaling

HSkMSC were starved for 3 h in Skeletal Muscle Cell Medium supplemented with 0.25% BSA and 1% Penicillin-Streptomycin. Medium supplemented with recombinant insulin (Actrapid, Novo Nordisk) (100 nM) or IGF-1 (Life technologies) (100 ng/ml, equivalent to 13 nM) was added to the cells for 20 min.

### 2.5 RNA extraction and gene expression analyses in cell lines and human biopsies

Total RNA from cells and biopsies was performed as previously described [31]. In short, RNA from cells was isolated using E.Z.N.A. total RNA kit according to the manufacturer's protocol. RNA was isolated from skeletal muscle samples using TRIzol reagent (Life Technologies), according to the manufacturer's protocol. The quality of RNA was assessed using a NanoDrop 1000 (Thermo Fisher Scientific). cDNA synthesis was performed using High-Capacity cDNA Reverse Transcription Kit (Thermo Fisher Scientific). The quantitative real-time PCR (qRT-PCR) was performed using TaqMan probes on a QuantStudio 6 Flex Real-Time PCR System (Thermo Fisher Scientific). Information about primers is shown in Table S4.

### 2.6 Protein extraction and western blot analysis

Total proteins from cells were extracted as previously described [31]. In short, cells were washed with ice-cold PBS and protein lysates were prepared using a lysis buffer (Cell Lysis Buffer II) with a protease inhibitor cocktail and phosphatase inhibitors. Protein content was determined using Pierce BCA Protein Assay Kit. Protein lysates were boiled with NuPAGE (Invitrogen) sample buffer supplemented with Dithiothreitol (DTT) and boiled for 5 min at 95 °C. Protein samples were loaded into NuPAGE 4–12% Bis-Tris Protein Gels for electrophoresis. Proteins were transferred using the Trans-Blot Turbo Transfer System (Bio-Rad) on the nitrocellulose membranes (Bio-Rad). Membranes were blocked with 5% milk in PBST. Membranes were incubated with primary antibodies diluted in 1% milk in PBST at 4 °C overnight. The membranes were probed with primary and secondary antibodies (Table S4). Proteins were visualized by enhanced chemiluminescence Clarity Max Western ECL Substrate (Bio-Rad) using the ChemiDoc Imaging System (Bio-Rad) and ImageJ software.

### 2.7 Immunocytochemistry

Immunohistochemistry was performed as previously described [31]. In short, cells grown on glass slides were treated as described above. Cells were fixed in 4% (v/v) phosphate-buffered formaldehyde for 15min and permeabilized in 0.2% Triton X-100 for 10min. Cells were then blocked with 5% FBS for 1h, followed by incubation with primary antibodies against p21, phospho-histone H2AX, and Ki67 overnight. Cells were then incubated with secondary antibody conjugated with Alexa Fluor 594 for 1h at room temperature, nuclei were stained with DAPI (D9542, Sigma–Aldrich) and the coverslip was mounted with fluorescence mounting medium (Invitrogen). Pictures were obtained using a Zeiss Axio Observer. Image analysis was performed using ImageJ (v1.8.0 National Institutes of Health, NIH).

### 2.8 MitoTracker staining

MitoTracker staining was performed as previously described [31]. In short, cells grown on glass slides were stained with 100 nM MitoTracker Red CMXRos (Thermo Fisher Scientific) for 30min at 37 °C, followed by nuclear staining with DAPI (D9542, Sigma–Aldrich). Cells were washed and fixed in 4% (v/v) phosphate-buffered formaldehyde for 15min at RT. Images were taken using the Zeiss Axio Observer. Image analysis was performed using ImageJ (v1.8.0 National Institutes of Health, NIH).

### 2.9 Statistical analysis

All values are presented as mean ± SEM. Data were tested for normality using appropriate test and appropriate parametric or non-parametric statistical tests were performed. For comparisons between two groups statistical significance was determined by paired Student's t-test and unpaired Student's t-test (parametric tests) or Wilcoxon Signed-Rank Test and Mann–Whitney U Test (non-parametric tests). For comparison between multiple groups statistical significance was determined by one-way ANOVA with Dunnett's multiple comparisons test or Kruskal–Wallis Test. To assess correlation between variables, Spearman's rank correlation test was used. Multivariable linear regression analysis was performed to account for the potential confounding effect of sex and chronological age on the differences between lean individuals and individuals with obesity. In the human biopsy data, some values were missing due to undetected measurements and were excluded from the analysis. All statistical analyses and graphs were performed with GraphPad Prism 10.0 software. Significance is indicated in the figures and p-value ≤0.05 was considered statistically significant.

## 3 Results

### 3.1 CS markers are increased in SkM in middle-aged people with obesity

CS is a conserved mechanism of SkM aging in both mice and humans [14] and obesity may accelerate the aging phenotype in SkM [6]. Thus, to assess the impact of both aging and obesity on expression of CS markers in SkM, we analyzed muscle biopsies from the *vastus lateralis* of 23 lean (BMI 24.5 ± 0.3 kg/m^2^) young (27 ± 0.8 years) individuals and 32 individuals with obesity (without diabetes) (BMI 35 ± 0.6 kg/m^2^) and middle-aged (49 ± 1.4 years) (Table S1). Metabolic assessments revealed that individuals with obesity exhibited metabolic dysfunctions, characterized by increased HOMA-IR (p < 0.0001), increased HbA1c (p < 0.0001), elevated levels of fasting insulin (p < 0.0001), leptin (p < 0.0001), free fatty acids (p < 0.0001) and reduced adiponectin (p < 0.0001) (Table S1).

**Figure 1:**
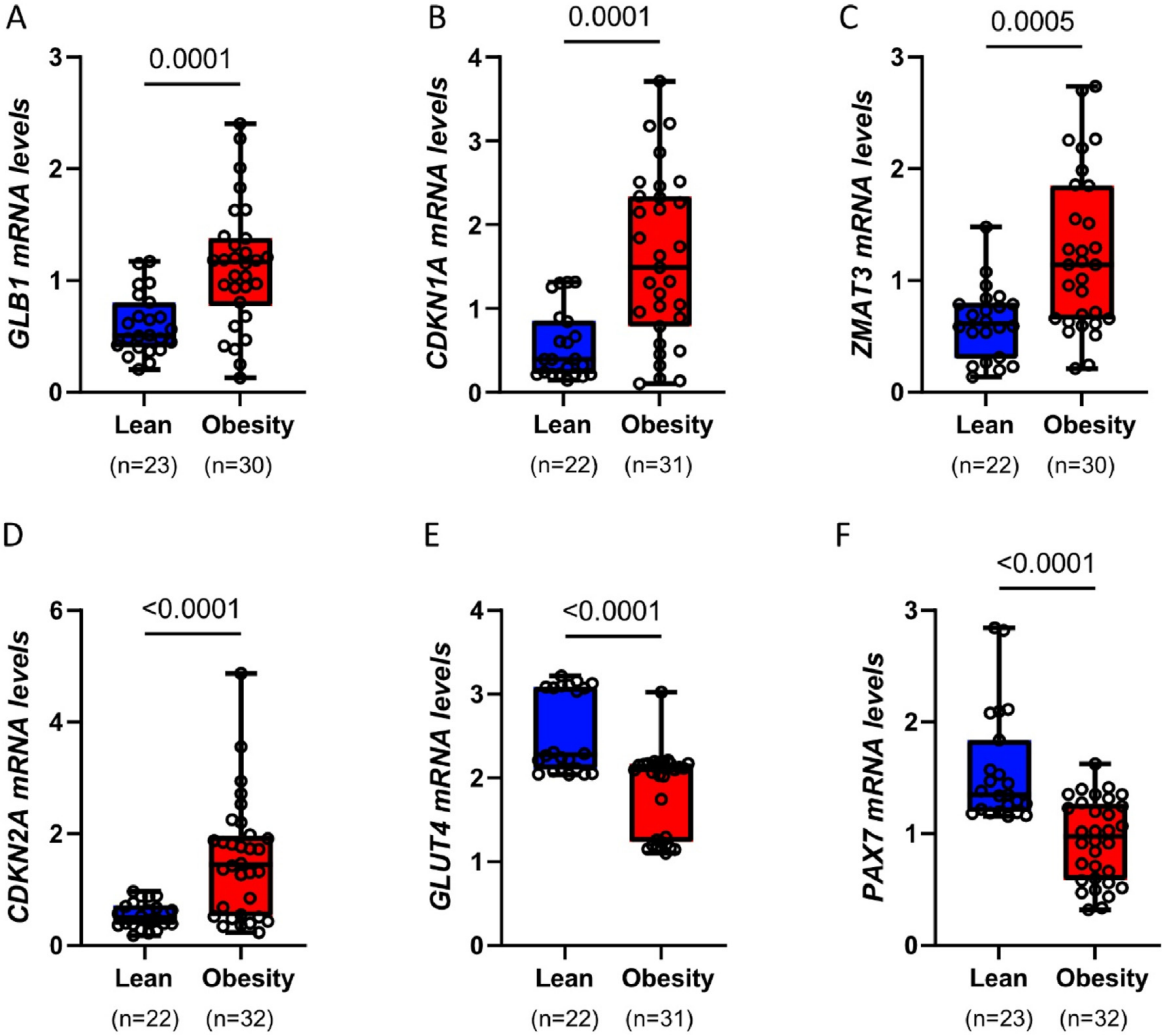
Subjects with obesity exhibited higher expression of senescence markers in SkM. (A–F) Bar graphs showing qRT-PCR analysis of the indicated genes, expressed as fold change and normalized to 18S. Data are presented as boxplots (min–max) with individual values shown. Normality of the data distribution was assessed using the Shapiro–Wilk test. Statistical comparisons were performed using an unpaired Student's t-test or Mann–Whitney test, with significance indicated on the graphs. Sample size varies between genes in the presented groups due to undetected values in qRT-PCR analysis. Undetected values were excluded to ensure reliable statistical evaluation.

To investigate CS, we analyzed the mRNA levels of canonical senescence markers [14]. Absolute quantification of mRNA levels from lean individuals and individuals with obesity revealed that the expression of senescence-associated beta galactosidase *(GLB1),* cyclin-dependent kinase inhibitor 1A *(CDKN1A),* zinc finger matrin-type 3 *(ZMAT3)* and cyclin-dependent kinase inhibitor 2A *(CDKN2A)* genes was significantly increased in SkM from middle-aged individuals with obesity compared to lean and young controls (Figure 1A–D). Given the reduced insulin sensitivity in the individuals with obesity, we also examined the mRNA levels of Glucose Transporter Type 4 (*GLUT4, also known as SLC2A4)* gene responsible for glucose uptake in SkM, which was significantly decreased in individuals with obesity (Figure 1E). Since the regenerative capacity of SkM declines with age [16], we further analyzed the expression of paired box protein 7 (PAX7), a transcription factor crucial for SkM development and regeneration [32]. *PAX7* expression was significantly decreased in individuals with obesity compared to lean controls (Figure 1F).

To investigate the association between changes in gene expression and metabolic parameters, we conducted a correlation analysis. This analysis revealed significant positive correlations between the mRNA expression of *GLB1, CDKN1A, ZMAT3* and *CDKN2A* with age, weight, BMI, fasting plasma levels of leptin and insulin, as well as glucose (OGTT) (Table S2). In contrast, we observed significant negative correlations between the mRNA expression of *GLUT4* and *PAX7* and these same parameters. Overall, our findings indicate that the expression of senescence-related genes in skeletal muscle is increased in middle-aged individuals with obesity and correlates with a dysmetabolic state.

To further understand the role of CS in the metabolic dysfunction seen in individuals with obesity, we assessed the correlation between senescence markers and genes with a key role in metabolism (Table S3). This analysis showed that canonical senescence markers were positively correlated with each other and negatively correlated with *PAX7* and *GLUT4*. The positive correlation between senescent markers suggests a consistent and coordinated activation of a senescent program in SkM, while a simultaneous negative correlation with *GLUT4* and *PAX7* implies an impact of CS on key metabolic and regulatory functions of SkM.

Taken together, our analysis reveals that SkM from middle-aged individuals with obesity exhibit increased CS markers which are associated with a dysmetabolic state, reduced expression of key metabolic gene *GLUT4* and diminished levels of *PAX7*, which is crucial for the maintenance and development of SkM.

Given the observed differences between lean individuals and individuals with obesity, we next sought to determine the extent to which these differences were influenced by age and sex—two well-established factors impacting both metabolic function and CS [14,[33], [34], [35]]. This adjustment was particularly important because the lean subjects and individuals with obesity were not well-balanced in terms of sex composition and age. These disparities raise the possibility that some of the observed differences in gene expression could be partially driven by age or sex rather than obesity *per se*. To address this, we performed multivariable linear regression analyses to adjust for sex alone, as well as for both sex and chronological age. Sex-adjusted analysis confirmed that obesity remains a primary driver of SkM senescence. Adjusting for sex alone did not alter the significant upregulation of *GLB1*, *ZMAT3*, *CDKN1A*, and *CDKN2A* in individuals with obesity (Sup. Figure 1A–D). Similarly, the downregulation of *GLUT4* and *PAX7* remained robustly significant (Sup. Figure 1E and F). These findings reinforce the notion that obesity exerts a direct influence on SkM senescence and metabolic impairment, independent of sex-related differences. Age-adjusted analysis further highlights the interplay between obesity and chronological aging in driving CS. When adjusting for both sex and chronological age, we observed that *GLB1* and *ZMAT3* expression remained significantly elevated in the subjects with obesity (Sup. Figure 2A and C), confirming that obesity independently contributes to increased senescent burden in SkM. However, *CDKN1A* exhibited only a trend toward upregulation, while *CDKN2A* was no longer significantly different between groups (Sup. Figure 2B and D). These findings indicate that age is a major factor influencing the expression of certain senescence-related genes, particularly *CDKN2A*. Importantly, despite adjusting for both age and sex, the expression of *GLUT4* and *PAX7* remained significantly downregulated in the individuals with obesity (Sup. Figure 2E and F), underscoring the persistent metabolic and regenerative impairments associated with obesity, independent of aging.

These results provide key insights into the interplay between age, sex, and obesity in modulating SkM senescence and metabolic function. While age is a significant determinant of CS burden, our findings demonstrate that obesity acts as an independent contributor, further exacerbating SkM dysfunction beyond age-related effects alone. The fact that the core pattern of obesity-induced dysregulation remains largely consistent after adjustment for sex and age underscores the profound impact of excess adiposity on muscle health.

### 3.2 Physical exercise reduces senescence markers in SkM cells of lean individuals

**Figure 2:**
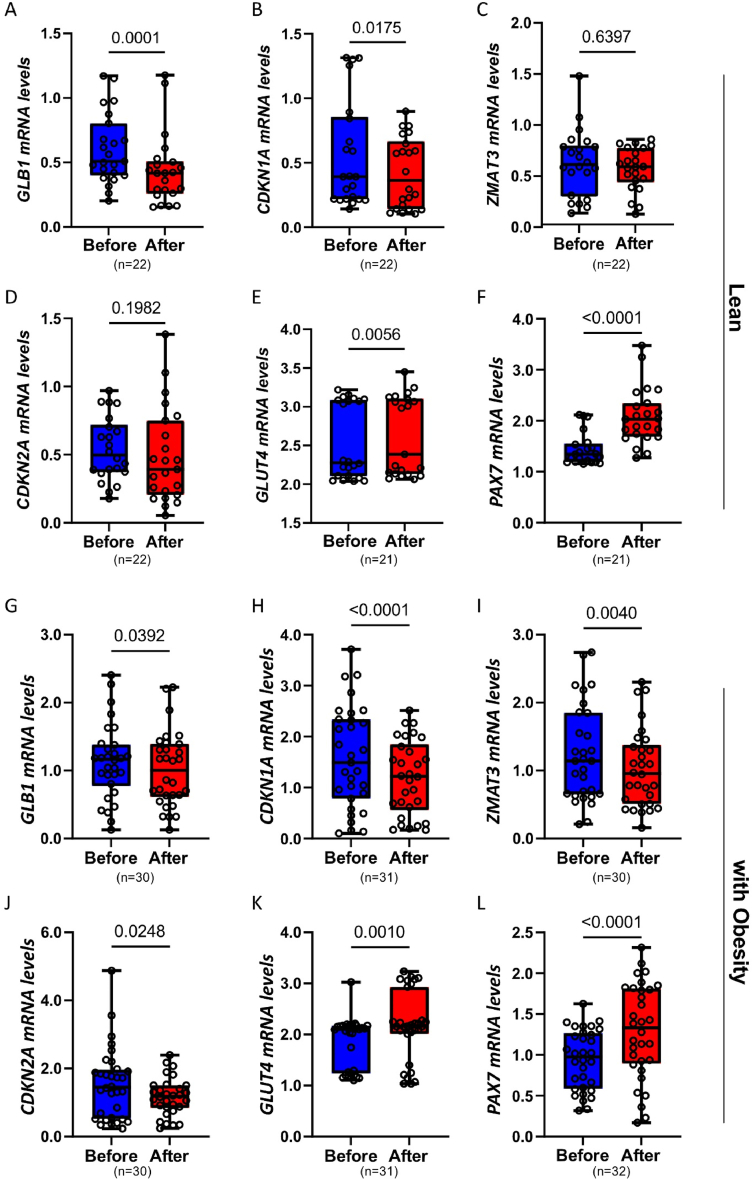
Impact of exercise intervention on gene expression in SkM. (A–F) qRT-PCR analysis of the indicated genes in 23 young, lean individuals before and one month after an exercise intervention. (G–L) qRT-PCR analysis of the indicated genes in 32 middle-aged individuals with obesity before and after a six-month exercise intervention. Data are presented as boxplots (min–max) with individual values displayed. Normality of the data distribution was assessed using the Shapiro–Wilk test. Statistical comparisons were performed using paired Student's t-test or Wilcoxon signed-rank test, with significance indicated on the graphs. Sample size varies between genes in the presented groups due to undetected values in qRT-PCR analysis. Undetected values were excluded to ensure reliable statistical evaluation.

Metabolic dysfunction and aging are linked to increased CS and impaired SkM remodeling, which may reduce the muscle's ability to adapt to exercise [14,36]. Thus, to investigate the effect of physical exercise on CS in human SkM, we compared the expression of senescence markers before and after a 4-week exercise program in the SkM biopsies obtained from the same 23 young (27 ± 0.8 years) and lean (BMI 24.5 ± 0.3 kg/m^2^) individuals shown in Table S1. Anthropometric and metabolic analyses revealed that 4 weeks of exercise significantly improved OGTT glucose (p = 0.0242), fasting insulin (p = 0.0084), HOMA-IR (p = 0.0058), glucose infusion rate in the steady state of an euglycemic-hyperinsulinemic clamp (p < 0.0001) and lipid metabolism parameters (HDL-C (p < 0.0001), triglycerides (p < 0.0001) and free fatty acids (p = 0.0005)) (Table 1), confirming the exercise regimen's efficacy in improving metabolic phenotype. Interestingly, we also found that *GLB1* and *CDKN1A* were significantly downregulated in SkM biopsies after exercise, indicating a beneficial role of exercise in reducing CS (Figure 2A–B). However, *ZMAT3* and *CDKN2A* levels did not change (Figure 2C–D). The reduction in CS markers observed after 4 weeks of exercise was accompanied by a significant increase in *GLUT4* mRNA expression (Figure 2E). To further analyze changes in SkM, we analyzed the expression of *PAX7.* Our analysis revealed a significant increase in *PAX7* mRNA expression following the exercise, suggesting enhanced activation of satellite cells and muscle repair (Figure 2F).

**Table 1:** Characteristics of lean cohort study participants; before and after one month exercise.

	Before	After	*P values*
	n = 23	n = 23	
**Biometric parameters**			
Age, years	27 ± 0.8	27 ± 0.8	
Weight, kg	79 ± 2.0	79 ± 2.0	*0.3538*
BMI, kg/m^2^	24.5 ± 0.3	24.5 ± 0.3	*0.8207*
Fat mass, kg	14.1 ± 1.3	16.4 ± 1.4	*0.0034*
OGTT 0h glucose, mmol/l	5.0 ± 0.2	4.4 ± 0.2	*0.0294*
OGTT 2h glucose, mmol/l	5.4 ± 0.3	4.8 ± 0.2	*0.0242*
FPI, pmol/l	12.3 ± 1.7	9.7 ± 1.2	*0.0084*
HOMA-IR	0.40 ± 0.06	0.27 ± 0.04	*0.0058*
Clamp, μmol/kg/min	109 ± 2.5	114 ± 2.3	<*0.0001*
VO2 max,ml/min	3849 ± 130	3906 ± 111	*0.3745*
VO2 max, ml/min/kg	48.8 ± 1.5	49.0 ± 1.5	*0.2241*
Leptin, ng/ml	4.0 ± 0.6	4.1 ± 0.5	*0.5028*
Adiponectin, μg/ml	13.9 ± 0.8	15.2 ± 0.8	*0.0563*
Total cholesterol, mmol/l	5.2 ± 0.1	5.2 ± 0.1	*0.6546*
LDL-C, mmol/l	3.0 ± 0.1	3.0 ± 0.1	*0.7655*
HDL-C, mmol/l	1.7 ± 0.1	1.8 ± 0.1	<*0.0001*
Triglyceride, mmol/l	1.3 ± 0.05	1.2 ± 0.04	<*0.0001*
FFA, mmol/l	0.15 ± 0.01	0.12 ± 0.01	*0.0005*
hsCrP, mg/dl	1.09 ± 0.1	1.03 ± 0.1	*0.1353*

Data are mean ± SEM.

Taken together, our findings demonstrate that 4 weeks of regular physical training in lean, young men significantly reduced the expression of senescence-related genes in SkM, which was associated with improved insulin sensitivity, enhanced lipid metabolism, and activation of satellite cells.

### 3.3 Prolonged physical exercise reduces senescence markers in SkM cells of middle-aged individual with obesity

Several factors, including age and BMI, influence exercise response [37,38]. Older individuals and those with obesity have generally favourable exercise-induced adaptations in insulin sensitivity and other cardiometabolic risk factors, but they frequently fail to reach levels seen in younger people, possibly due to body composition differences [37,38]. Thus, we sought to determine whether age and obesity could also impact the beneficial effects of exercise on CS markers in SkM. For this reason, we measured the expression of senescence markers in SkM biopsies from the 32 middle-aged individuals with obesity which were subjected to a similar exercise regimen as that of the lean individuals but with a longer duration (i.e., 6 months). Six months of supervised exercise led to significant improvements in anthropometric parameters (weight (p < 0.0001), BMI (p < 0.0001), fat mass (p < 0.0001)), and metabolic state as indicated by a reduced glucose result (OGTT (p < 0.0001)), decreased HOMA-IR (<0.0001), decrease in plasma levels of fasting insulin (p < 0.0001), leptin (p < 0.0001), and inflammation marker hsCRP (p < 0.0001), as well as an increase in adiponectin and HDL-C levels (p < 0.0001) (Table 2).

**Table 2:** Characteristics of cohort study participants with obesity; before and after six months exercise.

	Before	After	*P values*
	n = 32	n = 32	
**Biometric parameters**			
Male (n; %)	20; 62	20; 62	
Age, years	49 ± 1.4	49 ± 1.4	
Weight, kg	108 ± 3.3	105 ± 3.0	<*0.0001*
BMI, kg/m^2^	35 ± 0.6	34 ± 0.6	<*0.0001*
Fat mass, %	33 ± 0.6	31 ± 0.5	<*0.0001*
OGTT 0h glucose, mmol/l	5.6 ± 0.1	5.4 ± 0.1	*0.0004*
OGTT 2h glucose, mmol/l	8.6 ± 0.2	7.1 ± 0.2	<*0.0001*
FPI, pmol/l	80 ± 8.4	55 ± 6.2	<*0.0001*
HOMA-IR	3.3 ± 0.3	2.2 ± 0.2	<0.0001
Leptin, ng/ml	23 ± 1.2	20 ± 1.2	<*0.0001*
Adiponectin, μg/ml	7 ± 0.6	9 ± 0.6	<*0.0001*
Total cholesterol, mmol/l	5 ± 0.2	5 ± 0.2	*0.8865*
LDL-C, mmol/l	3 ± 0.2	3 ± 0.2	*0.4879*
HDL-C, mmol/l	1.2 ± 0.1	1.4 ± 0.1	<*0.0001*
Triglyceride, mmol/l	1.7 ± 0.2	1.4 ± 0.1	*0.0004*
FFA, mmol/l	0.6 ± 0.1	0.5 ± 0.1	*0.3895*
hsCrP, mg/dl	1.6 ± 0.1	1.1 ± 0.1	<*0.0001*

Data are mean ± SEM.

After confirming the efficacy of the exercise regimen in improving the metabolic phenotype, we examined CS markers in the SkM. mRNA levels of *GLB1, CDKN1A, ZMAT3* and *CDKN2A* were significantly reduced following exercise in this cohort (Figure 2G–J). This effect was accompanied by a significant increase in *GLUT4* and *PAX7* expression (Fig. 2K-L), suggesting improved insulin sensitivity and enhanced satellite cell activation in SkM, as observed in young and lean individuals. Our analysis, therefore, demonstrates that physical training is effective in reducing the expression of senescence-related genes in SkM in middle-aged individuals with obesity, leading to improvements in insulin sensitivity, lipid metabolism, and increased activation of SkM satellite cells.

### 3.4 Exposure of HSkMSC to doxorubicin induces senescence

**Figure 3:**
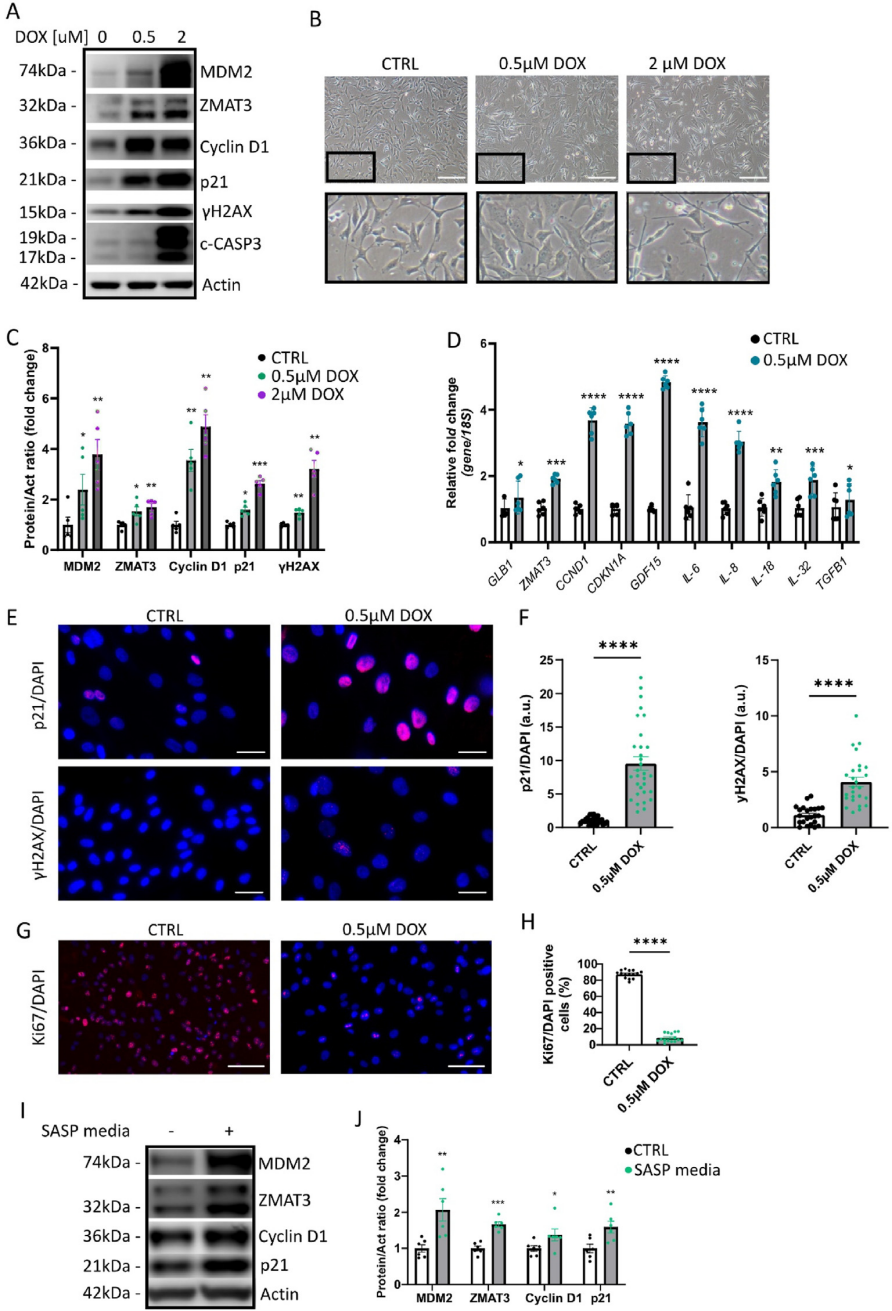
Doxorubicin exposure induces senescence in HSkMSC. (A) Representative immunoblots of the indicated proteins in control and DOX-treated cells after 48 h (n = 5). (B) Representative bright-field images of HSkMSC treated with varying concentrations of DOX for 48 h (n = 3). Scale bars = 60 μm. (C) Bar graphs showing relative protein levels in control and DOX-treated cells after 48 h, normalized to actin (n = 5). Data are presented as mean ± SEM, with individual data points shown as dots. (D) Bar graphs displaying qRT-PCR analysis of the indicated genes as fold change, normalized to 18S (n = 6). Data are presented as mean ± SEM, with dots representing individual data points. (E) Representative immunofluorescence images of control and DOX-treated cells stained for p21 (red) and nuclei (DAPI, blue), and γH2AX (red) (n = 3). Scale bars = 10 μm. (F) Bar graphs showing corrected total cell fluorescence of p21/DAPI and γH2AX/DAPI in control and DOX-treated cells (n = 3, with 4–6 randomly chosen fields per experiment). Dots represent individual data points. (G) Representative immunofluorescence images of control and DOX-treated cells stained for Ki67 (red) and nuclei (DAPI, blue) (n = 3). Scale bars = 25 μm. (H) Bar graphs showing the percentage of Ki67-positive cells relative to total nuclei in control and DOX-treated cells (n = 3, with 4–6 randomly chosen fields per experiment). Dots represent individual data points. (I) Representative immunoblots of the indicated proteins in control and SASP-positive media-treated cells (n = 6). (J) Bar graphs showing relative protein levels normalized to actin (n = 5). Normality of data distribution was assessed using the Shapiro–Wilk test. Depending on normality, either a paired Student's t-test (for normally distributed data) or a Wilcoxon matched-pairs test (for non-normally distributed data) was used. Data are presented as mean ± SEM. ∗p < 0.05, ∗∗p < 0.01, ∗∗∗p < 0.001, ∗∗∗∗p < 0.0001.

We observed that reduction in CS markers in SkM was associated with a marked increase in satellite cell activation, as indicated by *PAX7* upregulation following physical exercise. Given this relationship, we hypothesized that satellite cells may be directly influenced by the senescence processes. To test this, we examined the effects of doxorubicin, a well-established inducer of CS [39], specifically on satellite cells, assessing their activation and functional responses under these conditions. To determine the optimal concentration of DOX for inducing senescence, satellite cells were incubated with different concentrations of DOX for 2 h, followed by 48-hour incubation in fresh medium without DOX. A concentration of 2 μM DOX was too high, resulting in increased expression of cleaved caspase-3 (c-CASP3) (Figure 3A) and enhanced cell death (Figure 3B). On the other hand, 0.5 μM DOX did not induce c-CASP3, but led to CS as indicated by the enlarged and flattened cell morphology (Figure 3B) and upregulation of senescence markers MDM2, ZMAT3, Cyclin D1, p21 and γH2AX, as shown by western blot analysis (Figure 3A, C). The senescence phenotype was further confirmed by immunostaining for p21 and γH2AX (Figure 3E–F), as well as Ki67, a proliferation marker which was greatly reduced in senescent cells (Figure 3G–H). Based on this evidence, 0.5 μM DOX was selected for subsequent experiments. To further characterize the senescence phenotype, we analyzed gene expression of the above-mentioned senescence markers. RT-qPCR analysis showed that DOX increased the mRNA levels of *GLB1*, *ZMAT3, CCND1*, and *CDKN1A* (encoding p21) (Figure 3D). One hallmark of senescent cells is the acquisition of SASP phenotype [40]. Senescent cells secrete a whole spectrum of multiple factors such as cytokines, chemokines, growth factors and matrix-metalloproteinases that contribute to the senescent microenvironment [41]. To characterize the SASP in DOX-induced senescent satellite cells, we analyzed expression of several SASP genes and found significant upregulation of *Growth Differentiation Factor 15 (GDF15), Interleukin-6 (IL-6), Interleukin-8 (IL-8), Interleukin-32 (IL-32), Transforming Growth Factor Beta 1 (TGFB1)* in these cells (Figure 3D). Importantly, SASP can propagate senescence to neighboring healthy cells [42]. To verify this, we collected the SASP-enriched conditioned media from DOX-induced senescent satellite cells and applied it to naïve satellite cells. The induction of senescence in these recipient cells was confirmed by the increased expression of multiple senescence markers (Figure 3I–J).

To further examine the mechanism of senescence in SkM, we evaluated the pro-senescence effect of DOX on myoblasts (C2C12 cells). To determine the optimal concentration of DOX for inducing senescence in these cells, we incubated them with different DOX concentrations for 2 h, followed by incubation with fresh medium without DOX for 24 h. A concentration of 4 μM was too high, causing increased expression of c-Casp3 (Sup. Figure 3A), while 2 μM DOX was sufficient to induce senescence in murine myoblasts, as confirmed by upregulation of both senescence markers and SASP genes (Sup. Figure 3A–C). To verify whether also myocytes were sensitive to DOX-induced senescence, we differentiated the C2C12 myoblasts into myocytes. At day 5 of differentiation, Desmin differentiation marker was significantly upregulated (Sup. Figure 3D–E), as well as β subunit of insulin receptor (InsRβ) (Sup. Figure 3D–F) which is known to be upregulated during myoblast differentiation [43]. Importantly, 2 μM DOX was sufficient to induce senescence in differentiated myocytes, as indicated by upregulation of senescence markers p53, ZMAT3, Cyclin D1, p21 and γH2AX (Sup. Figure 3G–H).

In SkM, satellite cells are typically present in a quiescent state, and they become activated in response to muscle injury or stress. This activation leads to satellite cell proliferation and differentiation into myoblasts [44]. Thus, to assess the impact of CS on myoblast differentiation, we induced senescence by treating C2C12 cells with 2 μM DOX for 2 h, followed by a 24-hour incubation in fresh media. After this period, the cells were switched to differentiation medium to initiate the differentiation process. Our analysis revealed that by day 5 of differentiation, senescence markers remained significantly upregulated at both the protein and mRNA levels in DOX-treated cells (Sup. Figure 4A–C). However, the protein levels of differentiation markers Desmin and MyoD1, as well as *Myod1* mRNA, were significantly reduced in senescent cells (Sup. Figure 4A–C), indicating that CS impaired the myoblast differentiation.

Taken together, our findings demonstrate that DOX-induced senescent cells and their SASP impair SkM satellite cell function and myoblast differentiation. These findings highlight the detrimental effects of senescence on SkM function and regeneration.

### 3.5 CS impairs insulin signaling and reduces the expression of key muscle-regulatory genes

**Figure 4:**
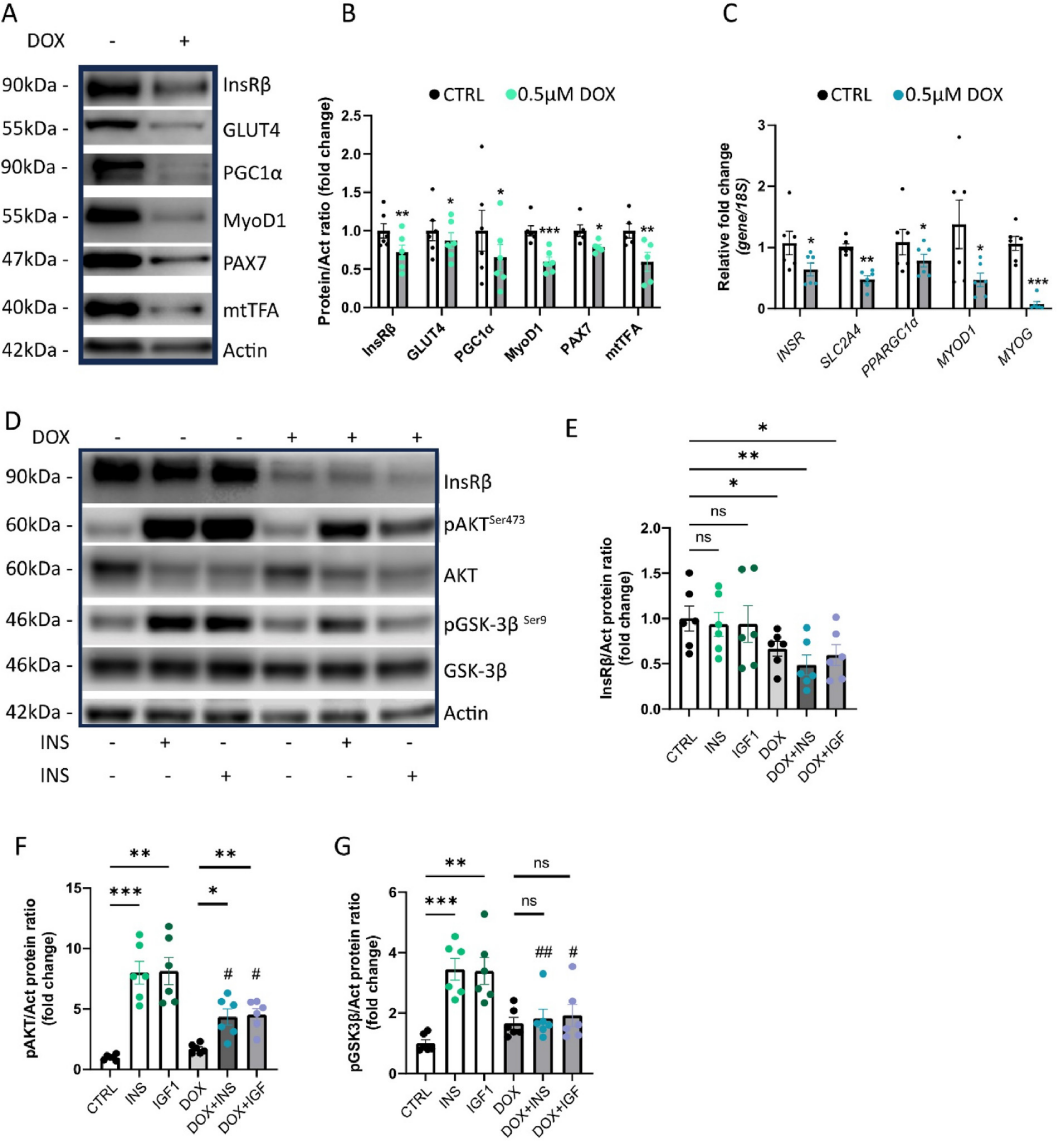
CS impairs insulin signaling and reduces the expression of key muscle-regulatory genes. (A) Representative immunoblots of the indicated proteins in control and DOX-treated cells after 48 h (n = 6). (B) Bar graphs showing relative protein levels in control and DOX-treated cells after 48 h, normalized to actin (n = 6). Data are presented as mean ± SEM, with individual data points shown as dots. (C) Bar graphs displaying qRT-PCR analysis of the indicated genes as fold change, normalized to 18S (n = 6). Data are presented as mean ± SEM, with dots representing individual data points. (D) Representative immunoblots of the indicated proteins in control and DOX-treated cells after 48 h, followed by exposure to insulin (INS) or IGF-1 for 20 min (n = 6). (E–G) Bar graphs showing relative protein levels in control and DOX-treated cells after 48 h, followed by exposure to INS or IGF-1 for 20 min, normalized to actin (n = 6). Data are presented as mean ± SEM, with dots representing individual data points. Normality of data distribution was assessed using the Shapiro–Wilk test. Depending on normality, either a paired Student's t-test (for normally distributed data) or a Wilcoxon matched-pairs test (for non-normally distributed data) was used. For comparisons involving multiple conditions, Repeated Measures One-Way ANOVA with Dunnett's multiple comparisons test (for normally distributed data) or the Friedman test (for non-normally distributed data) was applied. ∗p < 0.05, ∗∗p < 0.01, ∗∗∗p < 0.001 (control vs. treatment conditions); #p < 0.05, ##p < 0.01 (control + INS/IGF-1 vs. DOX + INS/IGF-1).

To further elucidate the impact of CS on satellite cells, we analyzed key regulatory proteins involved in insulin action and muscle function, including InsRβ and GLUT4. Both proteins were significantly reduced in DOX-induced senescent satellite cells (Figure 4A–B), which was also confirmed at mRNA levels (Figure 4C). To further investigate whether CS adversely affects satellite cells, we evaluated *PAX7* expression following DOX exposure and observed a significant downregulation (Figure 4A–B). Additionally, we examined the expression of genes involved in the molecular transduction of exercise-induced effects in SkM, such as peroxisome proliferator-activated receptor γ coactivator 1α (PGC1α; encoded by the *PPARGC1A* gene) [45], Myogenic Differentiation 1 (MyoD1) [46] and Myogenin (*MYOG*) in our *in vitro* model of senescent satellite cells. This analysis revealed that senescence in satellite cells leads to a reduction in PGC1α expression at both protein and mRNA levels (Figure 4A–C). Additionally, we found a significant decrease in the expression of MyoD1 (Figure 4A–C), along with reduced *MYOG* (Figure 4C). Given the negative impact of CS on PGC1α expression, we next investigated mitochondrial transcription factor A (mtTFA), a downstream regulator of mitochondrial function in SkM [47]. Our findings indicate that CS in satellite cells reduces the mtTFA expression (Figure 4A–B).

We also assessed the effects of CS on the insulin signaling pathway. After 3 h of serum starvation, senescent satellite cells were stimulated with acute insulin (INS, 100 nM). Insulin-stimulated phosphorylation of Protein kinase B/AKT (AKT) and Glycogen synthase kinase-3 beta (GSK3β) was significantly reduced in senescent satellite cells (Figure 4D–G). To reinforce these findings, we also subjected senescent satellite cells to acute IGF-1 (13 nM) stimulation which also was reduced. Importantly, senescent satellite cells displayed a marked downregulation of InsRβ expression (Figure 4D–E). Since both insulin and IGF-1 bind to InsRβ to initiate downstream signaling, these findings suggest that CS impairs the ability of satellite cells to activate the insulin signaling pathway in response to both stimuli, likely due to the reduced expression of InsRβ.

### 3.6 Senolytic agents Dasatinib and Quercetin target CS in satellite cells

**Figure 5:**
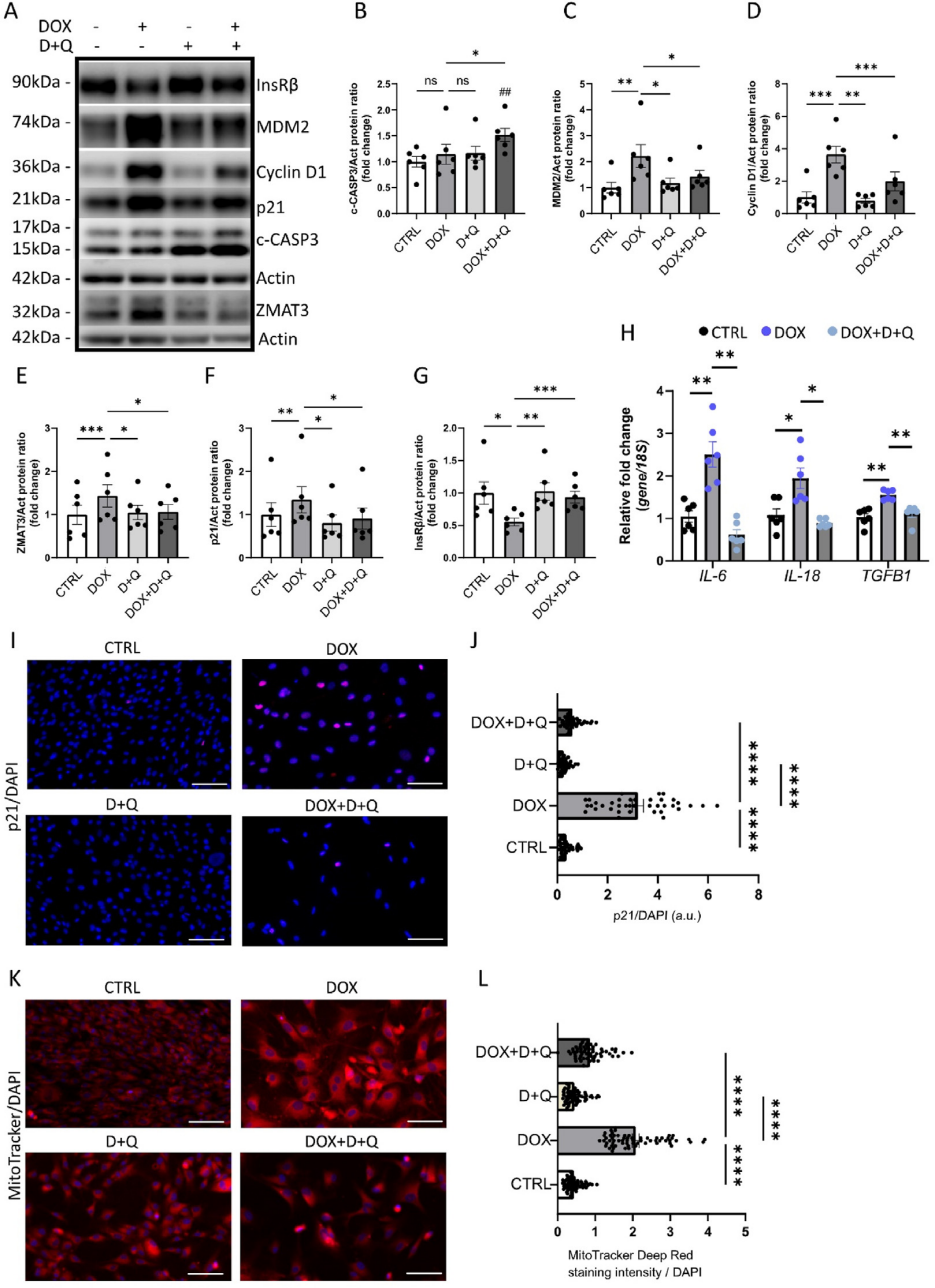
Senolytic agents Dasatinib and Quercetin target CS in satellite cells. (A) Representative immunoblots of the indicated proteins in control, DOX-, D + Q-, and DOX + D + Q-treated cells. (B–G) Bar graphs showing relative protein levels in the indicated groups, normalized to actin (n = 6). Data are presented as mean ± SEM, with dots representing individual data points. (H) Bar graphs displaying qRT-PCR analysis of the indicated genes as fold change, normalized to 18S (n = 6). Data are presented as mean ± SEM, with dots representing individual data points. (I) Representative immunofluorescence images of control, DOX-, D + Q-, and DOX + D + Q-treated cells stained for p21 (red) and nuclei (DAPI, blue) (n = 3). Scale bars represent 25 μm. (J) Bar graphs showing corrected total cell fluorescence of p21/DAPI in the indicated groups (n = 3, with 4–6 randomly chosen fields per experiment). (K) Representative immunofluorescence images of control, DOX-, D + Q-, and DOX + D + Q-treated cells stained with MitoTracker (red) and nuclei (DAPI, blue) (n = 3). Scale bars represent 25 μm. (L) Bar graph showing fluorescence intensities of MitoTracker Red, normalized to the number of nuclei (n = 3, with 4–6 randomly chosen fields per experiment). Dots represent individual data points. Normality of data distribution was assessed using the Shapiro–Wilk test. Depending on normality, either Repeated Measures One-Way ANOVA with Dunnett's multiple comparisons test (for normally distributed data) or the Friedman test (for non-normally distributed data) was applied to (B–H). For (J) and (L), One-Way ANOVA with a Kruskal–Wallis test and Dunn's multiple comparisons test was used. ∗p < 0.05, ∗∗p < 0.01, ∗∗∗p < 0.001, ##p < 0.01 (Repeated Measures One-Way ANOVA with Dunnett's multiple comparisons test or the Friedman test, as appropriate); ∗∗∗∗p < 0.001 (One-Way ANOVA with a Kruskal–Wallis test and Dunn's multiple comparisons test).

To explore potential therapeutic strategies for targeting cellular senescence in satellite cells, we investigated the efficacy of senolytic agents Dasatinib (D) and Quercetin (Q). These agents have previously demonstrated the ability to selectively eliminate senescent cells and improve muscle strength in aged mice by reducing CS in skeletal muscle [48,49]. We induced senescence in satellite cells by treating them with 0.5 μM DOX for 2 h, followed by 24-hour incubation in fresh media. After this, the senolytic cocktail (D: 200 nM and Q: 50 μM) was applied for 24 h, which resulted in increased apoptosis of senescent cells, as shown by elevated levels of c-CASP3 (Figure 5A–B). Notably, the significant increase in c-CASP3 expression was observed specifically in cells treated with both DOX and senolytics, and not in cells treated with senolytics alone. This was paralleled by a significant reduction of senescence markers MDM2, Cyclin D1, p21 and ZMAT3 (Figure 5A, C–F). The downregulation of p21 was also confirmed by immunostaining (Figure 5I–J). To further support the clearance of senescent cells by senolytics, we analyzed SASP gene expression and saw significant reductions in mRNA levels of *IL-6*, *IL-18*, and *TGFB1* following D + Q treatment (Fig. 5H).

We also demonstrated that D + Q treatment restored InsRβ levels in senescent cells (Figure 5A, G). Additionally, we assessed mitochondrial function by measuring mitochondrial membrane potential using MitoTracker Red. DOX-induced senescent satellite cells featured mitochondrial hyperpolarization (an excessive mitochondrial activity), as evidenced by increased fluorescence intensity (Figure 5K, L). Importantly, D + Q treatment normalized mitochondrial membrane potential, suggesting improved mitochondrial function (Figure 5K, L).

Taken together, our results demonstrate that the D + Q combination effectively eliminates senescent satellite cells by inducing apoptosis, leading to significant reductions of senescent markers and SASP genes. Importantly, this restored InsRβ levels and normalized mitochondrial function. These results highlight the therapeutic potential of D and Q as a senolytic strategy to mitigate the adverse effects of CS on SkM function.

### 3.7 An exercise mimetic mitigates the pro-senescence effect of doxorubicin while preserving its proinflammatory role, potentially supporting SkM regeneration

**Figure 6:**
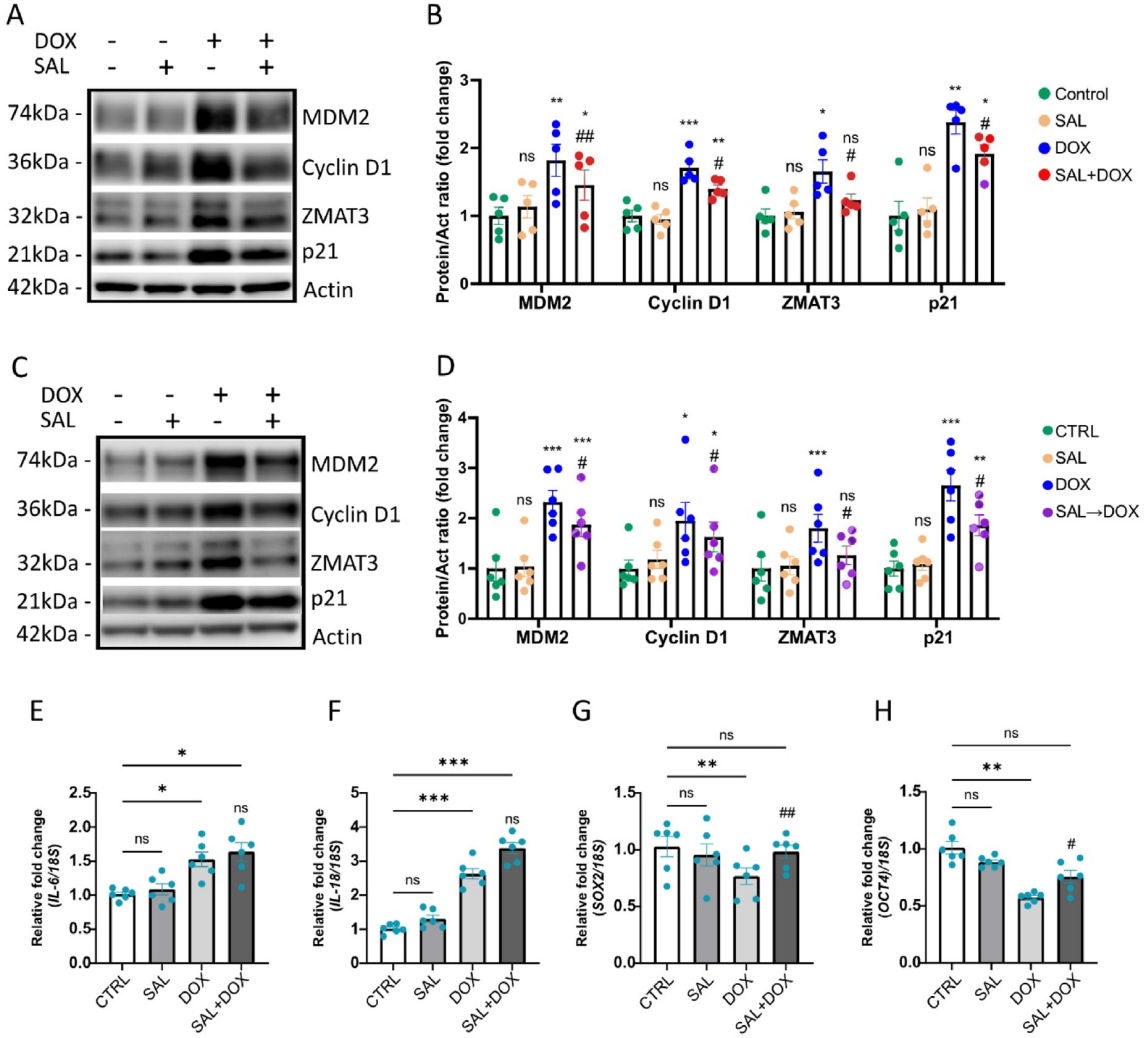
Salbutamol mitigates the pro-senescence effects of doxorubicin while preserving its proinflammatory role, which may support skeletal muscle regeneration. (A) Representative immunoblots of the indicated proteins in control, salbutamol-, DOX-, and salbutamol + DOX-treated cells after 24 h (n = 5). (B) Bar graphs showing relative protein levels in the indicated conditions after 24 h, normalized to actin (n = 5). Data are presented as mean ± SEM, with dots representing individual data points. (C) Representative immunoblots of the indicated proteins in control, salbutamol-, DOX-, and salbutamol pretreatment + DOX-treated cells after 24 h (n = 5). (D) Bar graphs showing relative protein levels in the indicated conditions after 24 h, normalized to actin (n = 5). (E–H) Bar graphs showing qRT-PCR analysis of the indicated genes, presented as fold change, normalized to 18S (n = 6). Data are presented as mean ± SEM, with dots representing individual data points. Normality of data distribution was assessed using the Shapiro–Wilk test. Depending on normality, either Repeated Measures One-Way ANOVA with Dunnett's multiple comparisons test (for normally distributed data) or the Friedman test (for non-normally distributed data) was applied. ∗p < 0.05, ∗∗p < 0.01, ∗∗∗p < 0.001 for control vs. treatments; #p < 0.05, ##p < 0.01 for DOX vs. SAL + DOX or DOX vs. SAL→DOX (Repeated Measures One-Way ANOVA with Dunnett's multiple comparisons test or the Friedman test, as appropriate).

Salbutamol, a β-adrenergic receptor agonist, is known to mimic some effects of exercise *in vitro* [50,51]. Thus, we examined the potential of salbutamol to modulate DOX-induced senescence in satellite cells. To this end, we employed two experimental approaches. First, we examined whether salbutamol counteracts DOX-induced senescence when administered simultaneously with DOX. Satellite cells were treated with DOX and salbutamol for 2 h, followed by incubation in fresh medium for 24 h. Western blot analysis revealed a significant reduction in senescence markers, including MDM2, Cyclin D1, ZMAT3 and p21, when salbutamol was co-administered with DOX (Figure 6A–B). To further understand the role of salbutamol, we pretreated satellite cells with salbutamol for 2 h, before exposure to DOX, followed by a 24-hour incubation in fresh medium. Remarkably, pretreatment with salbutamol also led to a significant reduction in the expression of senescence and cell cycle inhibition markers (Figure 6C–D). Taken together, these findings identify the novel cellular target of salbutamol and suggest that salbutamol effectively mitigates the pro-senescence effect of DOX in satellite cells, regardless of whether it is administered simultaneously or as a pretreatment.

To further characterize the mechanisms of salbutamol on DOX-induced senescence, we analyzed markers of SASP. While salbutamol reduced senescence markers, SASP components such as *IL-6* and *IL-18* did not significantly decrease following simultaneous treatment with DOX (Figure 6E–F). This differential effect on SASP production, between senolytics and salbutamol exposure, is likely due to the small inhibitory effect of salbutamol on p21 expression. Since p21 is a powerful signaling molecule and regulator of CS and SASP production in SkM [52], a stronger effect on the reduction of p21 might be required to reduce SASP genes. Additionally, this observation indicates that, unlike senolytic treatments, salbutamol may not impact SASP genes and have senomorphic effects. Importantly, given the established role of inflammation in SkM regeneration [53], the preservation of SASP markers may reflect a pro-regenerative effect exerted by salbutamol.

To elucidate the mechanism by which salbutamol reduces senescence and potentially supports muscle regeneration, we evaluated the expression of SRY-box transcription factor 2 (SOX2) and Octamer-binding transcription factor 4 (OCT4), key markers of pluripotency and stem cell self-renewal [54]. It was previously shown that expression of those markers induced activation of satellite cells, leading to increased muscle regeneration in mouse model [55]. Our results demonstrated that exposure to DOX significantly downregulated *SOX2* and *OCT4* expression (Figure 6G–H). Notably, this downregulation was counteracted by the simultaneous addition of salbutamol, suggesting that salbutamol may activate satellite cells even under cytostatic conditions (Figure 6G–H). In summary, our findings reveal that salbutamol mitigates the cytostatic effects of DOX while preserving its proinflammatory consequences. This dual action suggests that salbutamol may support SkM regeneration by reducing CSand promoting satellite cell activation, highlighting its potential as a therapeutic exercise mimetic in mitigating DOX-induced muscle damage.

## 4 Discussion

Aging is a key driver of CS, leading to the accumulation of senescent cells across tissues including SkM. This accumulation has detrimental effects on tissue function and metabolism [3,56]. In this study, we analyzed expression profiles in human SkM biopsies collected from young, lean individuals and middle-aged individuals with obesity, where we clearly found significant increases in senescence markers in relation to age and obesity. Moreover, correlation analysis showed a positive association between CS markers in SkM and both age and body weight, along with key metabolic parameters, suggesting that obesity and aging accelerate CS in SkM, compromising both metabolic health and muscle function. Our results highlight the critical relationship between CS and metabolic dysfunction, particularly insulin resistance and glucose intolerance, which are hallmark features of both aging and obesity. While age is a recognized determinant of CS, our findings provide strong evidence that obesity independently exacerbates the senescence burden, contributing to a further decline in SkM function beyond the effects of aging alone. This emphasizes the urgent need for targeted interventions addressing obesity to prevent or mitigate SkM dysfunction and its broader health consequences.

Metabolically, the accumulation of senescent cells in SkM contributes to insulin resistance, a hallmark of aging and obesity [14,57]. Senescent cells produce an altered secretory phenotype SASP, which includes pro-inflammatory cytokines, growth factors, and proteases. These factors can lead to local inflammation and disruption of normal muscle function [49]. Particularly, CS in satellite cells, which are essential for muscle regeneration, impairs the ability to activate and repair muscle tissue following injury or stress. This contributes to the progressive decline in muscle mass, strength, and metabolic function seen with aging and obesity [16,58]. As insulin resistance develops, glucose uptake is impaired, and metabolic dysfunction increases, worsening the effects of sarcopenia. Thus, SkM senescence is not merely a consequence of aging and obesity but a central driver of metabolic dysregulation and disease progression.

Physical exercise has been shown to mitigate the burden of senescent cells [59], reducing the accumulation of age-related senescent cells and improving metabolic function [60]. While previous studies have reported beneficial effects of exercise on reducing CS in various tissues, including reduction in senescence markers in CD3+ T cells [23], colon mucosa [24] and circulating lymphocytes [61], the impact of exercise on CS in SkM in man is largely unexplored. Some reports suggest that resistance training decreases specific senescence markers, such as p16INK4a [62], while others indicate no effect on SA-β-Gal following moderate exercise [63]. However, these studies often focus on single senescence markers rather than providing a comprehensive analysis. Our study extends these findings by demonstrating that exercise significantly reduces multiple CS markers in human SkM and enhances insulin sensitivity, reinforcing the concept that exercise is not only a metabolic regulator but also a potent senotherapeutic.

The importance of regular physical activity in preventing chronic diseases and maintaining healthy SkM is well-established [21]. Sedentary lifestyle leads to muscle dysfunction and accelerated aging [21,64,65]. In SkM, accumulation of senescent cells affects both muscle fibers and satellite cells [49]. Satellite cells play a crucial role in muscle regeneration by activating upon injury or exercise. However, prolonged quiescence or senescence of satellite cells impairs muscle repair and contributes to sarcopenia [66,67]. Senescent satellite cells show impaired regenerative capacity, contributing to the age-related decline in muscle mass and strength [58]. Our findings further demonstrate that senescent satellite cells downregulate key regulatory genes essential for insulin signaling and muscle integrity, directly impairing glucose uptake and insulin action. Importantly, we show that exercise counteracts these effects by preserving satellite cell function, promoting muscle regeneration, and restoring insulin sensitivity, emphasizing the critical interplay between senescence, insulin resistance, and exercise.

A key translational aspect of our study is the identification of ZMAT3 as a novel and functionally relevant marker of SkM senescence. ZMAT3 was recently identified as an early marker and mediator of senescence in adipose precursor cells [68], with increased expression in senescence-enriched adipose tissue of first-degree relatives (FDR) of individuals with T2D [68,69]. Our study further supports ZMAT3 as a novel biomarker for CSin SkM. We provide the first evidence that ZMAT3 is significantly upregulated in senescent SkM and correlates with age, BMI, and metabolic dysfunction. Importantly, *ZMAT3* expression in SkM was significantly correlated with other established senescence markers such as *GLB1, CDKN1A*, and *CDKN2A*. Additionally, we demonstrated that treatment with senolytics effectively cleared senescent cells and restored ZMAT3 expression in satellite cells, providing evidence that ZMAT3 is actively involved in SkM senescence. These findings suggest that ZMAT3 could serve as a valuable target for therapeutic interventions aimed at mitigating age- and obesity-related muscle dysfunction, particularly by restoring muscle regeneration and insulin sensitivity.

Our findings provide a paradigm shift in the understanding of exercise as a direct modulator of SkM senescence and metabolic health, both in young, lean individuals and in middle-aged individuals with obesity. Our study is the first comprehensive human investigation to demonstrate that exercise actively reduces CS markers in SkM and parallelly increases GLUT4 expression, thereby improving insulin sensitivity and glucose homeostasis. Consistently, we report that CS in satellite cells impairs key regulatory genes, such as insulin receptor beta subunit, leading to a disruption of insulin signaling pathways. Our findings also show that salbutamol directly targets satellite senescent cells in SkM, indicating that it may enhance metabolic function not only by stimulating β2-adrenergic receptors but also by alleviating cellular senescence and its inflammatory secretome. In conclusion, our study underscores the fundamental role of cellular senescence in SkM metabolic dysfunction and position exercise as a potent non-pharmacological intervention for preserving muscle function and metabolic health. Moreover, the identification of ZMAT3 as a novel senescence marker provides a potential valuable tool for future research and clinical applications aimed at targeting SkM senescence to prevent age- and obesity-related metabolic diseases.

### 4.1 Study limitations

One limitation of this study is that the lean and obese subject groups were not perfectly age-matched, with the lean group having an average age of 27 years, while the obesity group had an average age of 49 years. The observed increase in CS markers in middle-aged subjects with obesity compared to younger, lean individuals is likely influenced by both age and body weight. Future studies are needed to investigate these findings in age-matched cohorts to better isolate the specific contributions of obesity and aging to cellular senescence.

Additionally, the two groups were not matched for the duration of exercise intervention. The lean group underwent a four-week physical training program, while the obesity group completed a six-month training program. As a result, direct comparisons between the groups could only be made at baseline (before exercise) and not after the intervention. However, we considered the longer study in older individuals with obesity as a “discovery” cohort to explore the effects of exercise on CS markers, while the younger, lean cohort served as an independent validation group to assess whether similar effects could be observed in a different population over a shorter timeframe. Furthermore, the study design was constrained by ethical considerations, as our Ethics Committee did not permit sequential muscle biopsies beyond two time points. This prevented the inclusion of an additional biopsy after four weeks in the obesity group and the extension of the lean group's intervention to six months. These regulatory restrictions ultimately shaped the selection of the two exercise protocols used in our study.

Another limitation is that we were only able to analyze gene expression changes at the mRNA level due to the limited amount of skeletal muscle tissue obtained through fine needle biopsies. We acknowledge that measurements of protein expression and phosphorylation would have further strengthened our findings on chronic changes in key genes related to cellular senescence and insulin resistance in skeletal muscle. However, given the small sample size of biopsy material, we were unable to perform Western blot analyses. Additionally, we intentionally collected biopsies 48–72 h after the final exercise session to capture more sustained gene expression changes while minimizing the impact of acute transcriptional changes. However, the absence of earlier time points prevents us from fully excluding transient transcriptional changes that may have occurred immediately post-exercise and potentially influenced the observed gene expression patterns. This approach aligns with recommendations from previous studies [27], and was approved by our Ethics Committee.

## CRediT authorship contribution statement

**Agnieszka Podraza-Farhanieh:** Writing – review & editing, Writing – original draft, Visualization, Validation, Methodology, Investigation, Formal analysis, Data curation, Conceptualization. **Rosa Spinelli:** Writing – review & editing, Visualization, Validation, Supervision, Methodology, Investigation, Formal analysis, Data curation, Conceptualization. **Federica Zatterale:** Writing – review & editing. **Annika Nerstedt:** Writing – review & editing. **Silvia Gogg:** Writing – review & editing. **Matthias Blüher:** Writing – review & editing, Methodology, Investigation. **Ulf Smith:** Writing – review & editing, Supervision, Resources, Project administration, Funding acquisition, Data curation, Conceptualization.

## Ethics statement

The study was approved by the Ethics Committee of the University of Leipzig (approval numbers: 030-2006; 031-2006; 159-12-21052012) and was performed in accordance with the Declaration of Helsinki. All participants gave written informed consent before taking part in the study and were informed of the purpose, risks and benefits of the investigation.

## Funding information

We acknowledge financial support from the Knut and Alice Wallenberg Foundation (2020.0118), the Novo Nordisk Foundation (NNF23OC0084066) and the Swedish Diabetes Research Foundation (DIA2024-884).

## Declaration of competing interest

The authors declare no competing interests.

## Data Availability

Data will be made available on request.
