# Supplementary material for: Physical training reduces cell senescence and associated insulin resistance in skeletal muscle
Source: Mol Metab. 2025 Mar 22;95:102130. doi: 10.1016/j.molmet.2025.102130 (PMC11994356; doi:10.1016/j.molmet.2025.102130)
Supplement: Multimedia component 1 [file mmc1.docx]

Supplementary material

|  | Lean | With Obesity | ***P values*** |
| --- | --- | --- | --- |
|  | n = 23 | n = 32 |  |
| **Biometric parameters** |  |  |  |
| Male (n; %) | 23;100 | 20; 62 | *0.0006* |
| Age, years | 27 ± 0.8 | 49 ± 1.4 | *<0.0001* |
| Weight, kg | 79 ± 2.0 | 108 ± 3.3 | *<0.0001* |
| BMI, kg/m² | 24.5 ± 0.3 | 35 ± 0.6 | *<0.0001* |
| Fat mass, % | 14.1 ± 1.3 | 33 ± 0.6 | *<0.0001* |
| OGTT 0h glucose, mmol/l | 5.0 ± 0.2 | 5.6 ± 0.1 | *0.0354* |
| OGTT 2h glucose, mmol/l | 5.4 ± 0.3 | 8.6 ± 0.2 | *<0.0001* |
| FPI, pmol/l | 12.3 ± 1.7 | 80 ± 8.4 | *<0.0001* |
| HOMA-IR | 0.40 ± 0.06 | 3.3 ± 0.3 | *<0.0001* |
| Hb1Ac, % | 5.2±0.2 | 5.9±0.2 | *<0.0001* |
| Adiponectin, µg/ml | 13.9 ± 0.8 | 7.1 ± 0.6 | *<0.0001* |
| Leptin, ng/ml | 4.0 ± 0.6 | 23 ± 1.2 | *<0.0001* |
| FFA, mmol/l | 0.15 ± 0.01 | 0.6 ± 0.1 | *<0.0001* |
| Total cholesterol, mmol/l | 5.2 ± 0.1 | 5.1 ± 0.2 | *0.8890* |
| LDL-C, mmol/l | 3.0 ± 0.1 | 3 ± 0.2 | *0.1643* |
| HDL-C, mmol/l | 1.7 ± 0.1 | 1.2 ± 0.1 | *0.0005* |
| Triglyceride, mmol/l | 1.3 ± 0.05 | 1.7 ± 0.2 | *0.1979* |
| hsCrP, mg/dl | 1.09 ± 0.1 | 1.6 ± 0.1 | *0.1008* |
| Data are mean ± SEM |  |  |  |

Table S1. Characteristics of cohort study participants

|  | *ZMAT3 mRNA level* | | |  | *GLB1 mRNA level* | | |  | *CDKN1A mRNA level* | | |  | *CDKN2A mRNA level* | | |  | *GLUT4 mRNA level* | | |  | | *PAX7 mRNA level* | | | |
| --- | --- | --- | --- | --- | --- | --- | --- | --- | --- | --- | --- | --- | --- | --- | --- | --- | --- | --- | --- | --- | --- | --- | --- | --- | --- |
|  | p value | R (Correlation Coefficient) | N |  | p value | R (Correlation Coefficient) | N |  | p value | R (Correlation Coefficient) | N |  | p value | R (Correlation Coefficient) | N |  | p value | R (Correlation Coefficient) | N |  | p value | | R (Correlation Coefficient) | N |  |
| Age. years | ***0.0046*** | 0.3873 | 52 |  | ***0.0006*** | 0.4561 | 53 |  | ***0.0003*** | 0.4824 | 53 |  | ***0.0009*** | 0.4339 | 55 |  | ***0.0007*** | -0.4510 | 53 |  | ***0.0002*** | | -0.4863 | 55 |  |
| BMI. kg/m² | ***0.0001*** | 0.502 | 52 |  | ***0.0005*** | 0.4619 | 53 |  | ***0.0003*** | 0.4762 | 53 |  | ***0.0052*** | 0.3718 | 55 |  | ***0.0002*** | -0.4935 | 53 |  | ***<0.0001*** | | -0.6284 | 55 |  |
| Weight. kg | ***0.0007*** | 0.4552 | 52 |  | ***0.0317*** | 0.2955 | 53 |  | ***<0.0001*** | 0.5393 | 53 |  | ***0.0052*** | 0.3718 | 55 |  | ***0.0009*** | -0.4439 | 53 |  | ***<0.0001*** | | -0.6268 | 55 |  |
| Leptin. ng/ml | ***0.0001*** | 0.5104 | 52 |  | ***0.0118*** | 0.3435 | 53 |  | ***0.0189*** | 0.3214 | 53 |  | ***0.0050*** | 0.3694 | 55 |  | ***0.0100*** | -0.3510 | 53 |  | ***0.0008*** | | -0.4380 | 55 |  |
| OGTT (2h). h | ***0.0053*** | 0.3813 | 52 |  | ***0.0010*** | 0.4407 | 53 |  | ***0.0047*** | 0.3823 | 53 |  | ***0.0040*** | 0.3821 | 55 |  | ***0.0579*** | -0.2622 | 53 |  | ***0.0020*** | | -0.4078 | 55 |  |
| FPI. pmol/l | ***0.0054*** | 0.3807 | 52 |  | ***0.0239*** | 0.3099 | 53 |  | ***0.0070*** | 0.3661 | 53 |  | ***0.0068*** | 0.3609 | 55 |  | ***0.0021*** | -0.4128 | 53 |  | ***0.0022*** | | -0.4047 | 55 |  |

Table S2. Spearman correlation analysis between skeletal muscle genes and metabolic parameters. Sample size (N) varies between genes due to undetected values in qRT-PCR analysis. Undetected values were excluded to ensure reliable statistical evaluation.

|  | *ZMAT3 mRNA level* | | |  | | *GLB1 mRNA level* | | | |  | | *CDKN1A mRNA level* | | | |  | | *CDKN2A mRNA level* | | | |  | | *PAX7 mRNA level* | | | | |
| --- | --- | --- | --- | --- | --- | --- | --- | --- | --- | --- | --- | --- | --- | --- | --- | --- | --- | --- | --- | --- | --- | --- | --- | --- | --- | --- | --- | --- |
|  | p value | R (Correlation Coefficient) | N | |  | | p value | R (Correlation Coefficient) | N | |  | | p value | R (Correlation Coefficient) | N | |  | | p value | R (Correlation Coefficient) | N | |  | | p value | R (Correlation Coefficient) | N |  |
| *ZMAT3 mRNA level* |  |  |  | |  | |  |  |  | |  | |  |  |  | |  | |  |  |  | |  | |  |  |  |  |
| *GLB1 mRNA level* | ***0.0028*** | 0.4139 | 50 | |  | |  |  |  | |  | |  |  |  | |  | |  |  |  | |  | |  |  |  |  |
| *CDKN1A mRNA level* | ***0.0386*** | 0.2934 | 50 | |  | | *0.1706* | 0.1948 | 51 | |  | |  |  |  | |  | |  |  |  | |  | |  |  |  |  |
| *CDKN2A mRNA level* | *0.3813* | 0.1240 | 52 | |  | | *0.093* | 0.2331 | 53 | |  | | ***<0.0001*** | 0.5143 | 53 | |  | |  |  |  | |  | |  |  |  |  |
| *PAX7 mRNA level* | ***0.0226*** | -0.3156 | 52 | |  | | ***0.0017*** | -0.4216 | 53 | |  | | ***0.0025*** | -0.4069 | 53 | |  | | *0.1153* | -0.2148 | 55 | |  | |  |  |  |  |
| *GLUT4 mRNA level* | *0.0662* | -0.2618 | 50 | |  | | ***0.0051*** | -0.3867 | 51 | |  | | ***<0.0001*** | -0.5389 | 51 | |  | | ***0.0078*** | -0.3616 | 53 | |  | | ***0.0201*** | 0.3185 | 53 |  |

Table S3. Spearman correlation analysis between skeletal muscle genes. Sample size (N) varies between genes due to undetected values in qRT-PCR analysis. Undetected values were excluded to ensure reliable statistical evaluation.

| REAGENT or RESOURCE | SOURCE | IDENTIFIER |  |
| --- | --- | --- | --- |
|  |  |  |  |
|  |  |  |  |
| **Antibodies** |  |  |  |
| Rabbit monoclonal anti-MDM2 | Cell Signaling Technology | 86934 |  |
| Rabbit monoclonal anti- CCND1 | Cell Signaling Technology | 55506 |  |
| Mouse monoclonal anti-phospho-histone H2A.X (Ser139) | Cell Signaling Technology | 80132 |  |
| Rabbit monoclonal anti-p53 | Cell Signaling Technology | 2527 |  |
| Rabbit monoclonal anti-cleaved Caspase 3 | Cell Signaling Technology | 9664s; RRID: AB_2070042 |  |
| Rabbit monoclonal anti-GLUT4 | Gift from K Stenkula, |  |  |
|  | Lund University, Sweden |  |  |
| Rabbit monoclonal anti-phospho-AKT (Ser473) | Cell Signaling Technology | 9271s |  |
| Rabbit monoclonal anti-AKT | Cell Signaling Technology | 9272s |  |
| Rabbit monoclonal anti-phospho-GSK3β | Cell Signaling Technology | 9331s |  |
| Mouse monoclonal anti-GSK3β | BD Biosciences | 610202; RRID: AB_397601 |  |
| Mouse monoclonal anti-Wig 1 (ZMAT3) | Santa Cruz Biotechnology | sc-398712 |  |
| Mouse monoclonal anti-p21 | Santa Cruz Biotechnology | sc-6246; RRID: AB_628073 |  |
| Mouse monoclonal anti-InsRβ | Santa Cruz Biotechnology | sc-57342; RRID: AB_784102 |  |
| Mouse monoclonal anti-PAX-7 | Santa Cruz Biotechnology | sc-81648 |  |
| Mouse monoclonal anti-mtTFA | Santa Cruz Biotechnology | sc-166965 |  |
| Mouse monoclonal anti-MyoD | Santa Cruz Biotechnology | sc-377460 |  |
| Mouse monoclonal anti-PGC1a | Santa Cruz Biotechnology | sc-518025 |  |
| Mouse monoclonal anti-Desmin | Santa Cruz Biotechnology | sc-23879 |  |
| Mouse monoclonal anti-Actin | Santa Cruz Biotechnology | sc-8432 |  |
| Mouse monoclonal anti-Ki67 | Cell Signaling Technology | 9449 |  |
| Anti-mouse IgG, HRP-linked Antibody | Cell Signaling Technology | 7076s |  |
| Anti-rabbit IgG, HRP-linked Antibody | Cell Signaling Technology | 7074s |  |
| Alexa Fluor 594 | Thermo Fisher Scientific | A-11005 |  |
| **Primers for qPCR** |  |  |  |
| GLB1 | Thermo Fisher Scientific | Hs01035168_m1 |  |
| ZMAT3 | Thermo Fisher Scientific | Hs00536976_m1 |  |
| CDNK1A | Thermo Fisher Scientific | Hs 00355782_m1 |  |
| CDNK2A | Thermo Fisher Scientific | Hs00923894_m1 |  |
| GDF15 | Thermo Fisher Scientific | Hs00171132_m1 |  |
| IL-6 | Thermo Fisher Scientific | Hs00174131_m1 |  |
| CXCL8 | Thermo Fisher Scientific | Hs00174103_m1 |  |
| IL-18 | Thermo Fisher Scientific | Hs00155517_m1 |  |
| IL-32 | Thermo Fisher Scientific | Hs00992441_m1 |  |
| TGFB1 | Thermo Fisher Scientific | Hs00171257_m1 |  |
| INSR | Thermo Fisher Scientific | Hs00961554_m1 |  |
| SLC2A4 (GLUT4) | Thermo Fisher Scientific | Hs00168966_m1 |  |
| PPARGC1a | Thermo Fisher Scientific | [Hs00173304_m1](https://www.thermofisher.com/taqman-gene-expression/product/Hs00173304_m1?CID=&ICID=&subtype=) |  |
| MYOD1 | Thermo Fisher Scientific | Hs00159528_m1 |  |
| SOX2 | Thermo Fisher Scientific | Hs01053049_s1 |  |
| Zmat3 | Thermo Fisher Scientific | Mm01292424_m1 |  |
| Cdkn1a | Thermo Fisher Scientific | Mm00432448_m1 |  |
| Ccnd1 | Thermo Fisher Scientific | Mm00432359_m1 |  |
| Bgal | Thermo Fisher Scientific | Mm00515342_m1 |  |
| Il-6 | Thermo Fisher Scientific | Mm00446190_m1 |  |
| Gdf15 | Thermo Fisher Scientific | Mm00442228_m1 |  |
| Myod1 | Thermo Fisher Scientific | Mm00440387_m1 |  |
| **Reagents** |  |  |  |
| Human Skeletal Muscle Satellite Cells (HSkMSC) | Innoprot | P10976 |  |
| Skeletal Muscle Cell Medium | Innoprot | P60124 |  |
| Murine Myoblast (C2C12) Cells | ATCC |  |  |
| Dulbecco's Modified Eagle Medium (DMEM) | Gibco | 11966-025 |  |
| Fetal Bovine Serum (FBS) | Gibco | 10270-106 |  |
| L-Glutamine | Gibco | 25030-024 |  |
| Penicillin-Streptomycin | Gibco | 15140-122 |  |
| Trypsin-EDTA | Gibco | 25300-054 |  |
| Mycoalert Mycoplasma Detection Kit | Lonza | LT07-318 |  |
| Recombinant Human Insulin | Actrapid_Novo Nordisk | 8-0204-49 |  |
| IGF1 | Life technologies |  |  |
| Bovine Serum Albumin (BSA Protein) | Sigma Aldrich | A3294 |  |
| DAPI Staining Solution | Sigma Aldrich | D9542 |  |
| Triton X-100 | Thermo Fisher Scientific | T8787 |  |
| ProLong™ Diamond Antifade Mountant | Invitrogen | P36970 |  |
| MitoTracker Red CMXRos | Invitrogen | M7512 |  |
| NuPAGE LDS Sample Buffer (4X) | Invitrogen | NP0007 |  |
| Cell Lysis Buffer II | Invitrogen | FNN0021 |  |
| Clarity Western ECL Blotting Substrate | Bio-Rad | 170-5060 |  |
| Restore Plus Western Blot Stripping Buffer | Thermo Fisher Scientific | 46430 |  |
| Doxorubicin | Sigma-Aldrich | 5040420001 |  |
| Dasatinib | Sigma-Aldrich | SML2589 |  |
| Quercetin | Sigma-Aldrich | Q4951 |  |
| Salbutamol | Sigma-Aldrich | S8260 |  |
| **Assays** |  |  |  |
| E.Z.N.A. total RNA kit | Omega Bio-tek | R6834-02 |  |
| High-Capacity cDNA Reverse Transcription Kit | Thermo Fisher Scientific | 4368814 |  |
| TaqMan Fast Advanced Master Mix | Applied Biosytems | 4444557 |  |
| Pierce BCA Protein Assay Kit | Thermo Fisher Scientific | 23225 |  |
| NuPAGE 4-12% Bis-Tris Protein Gels | Thermo Fisher Scientific | NP0336BOX |  |

Table S4. List of Reagents and Resources

Supplementary Figures


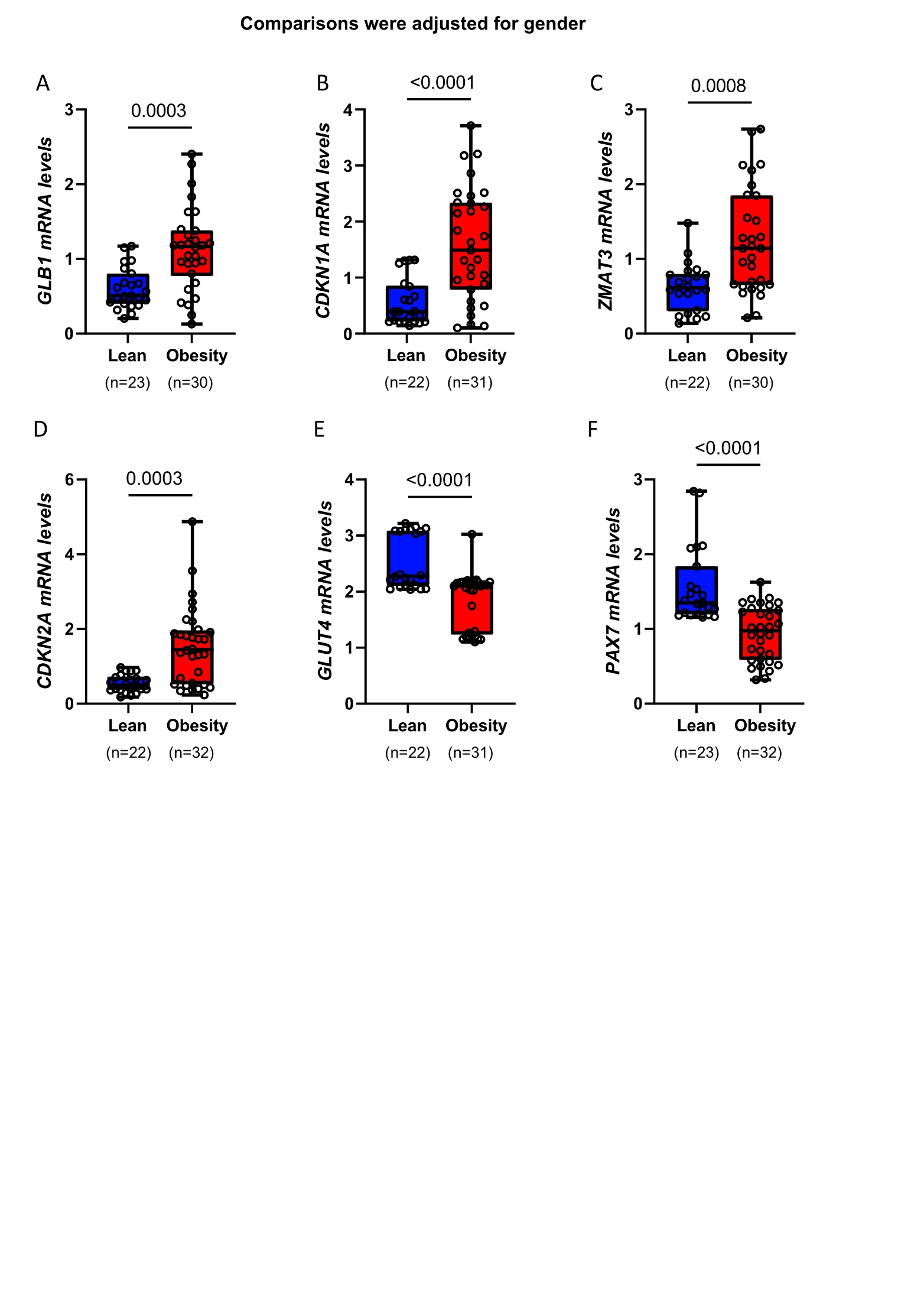


Sup. Fig 1.

(A-F) Bar graphs showing qRT-PCR analysis of the indicated genes, expressed as fold change and normalized to 18S. Data are presented as boxplots (min-max) with individual values shown. Normality of the data distribution was assessed using the Shapiro-Wilk test. *p* values shown on the graphs are adjusted for sex using multivariable linear regression analysis.


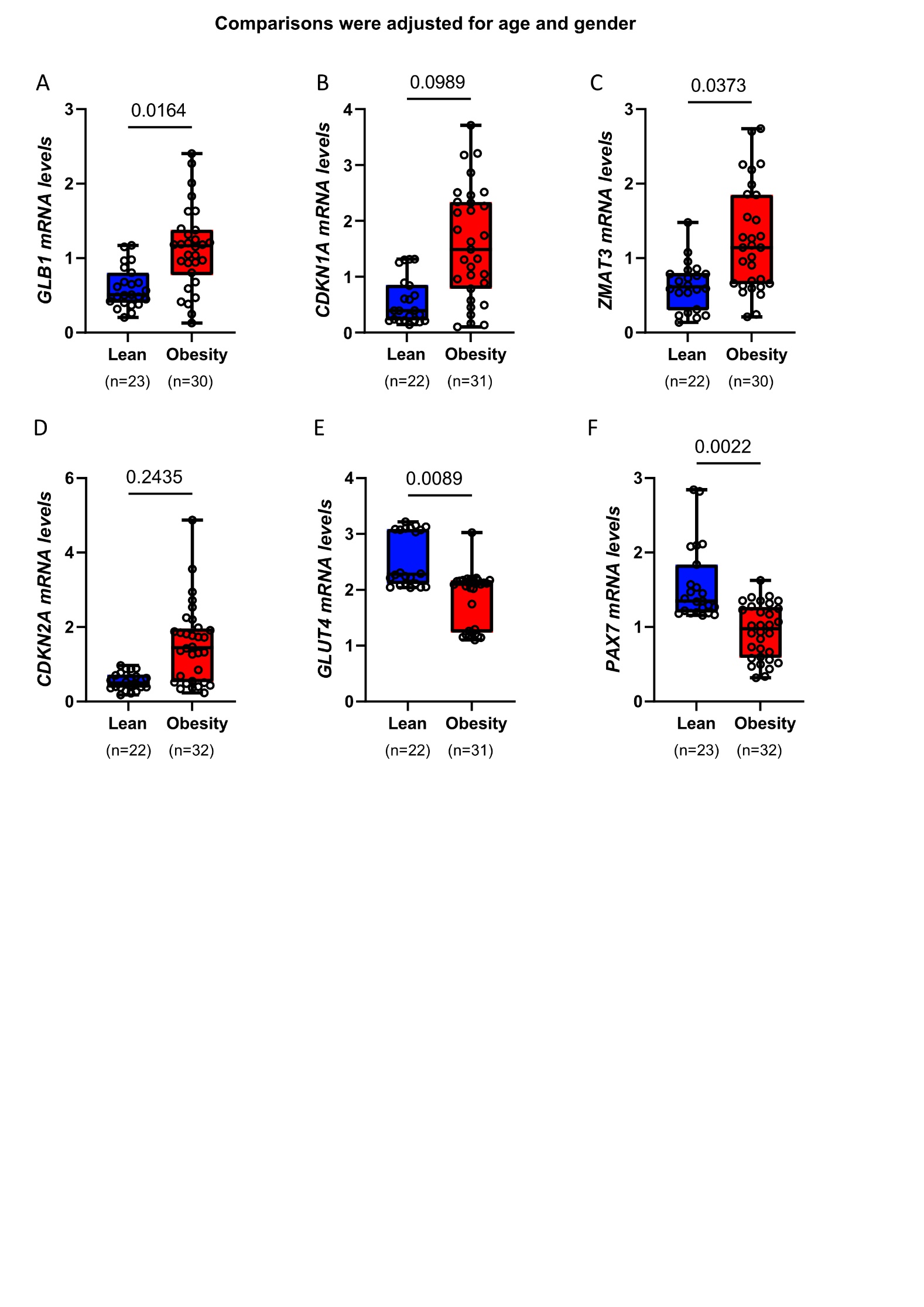


Sup. Fig 2.

(A-F) Bar graphs showing qRT-PCR analysis of the indicated genes, expressed as fold change and normalized to 18S. Data are presented as boxplots (min-max) with individual values shown. Normality of the data distribution was assessed using the Shapiro-Wilk test. *p* values shown on the graphs are adjusted for both sex and age presence using multivariable linear regression analysis.


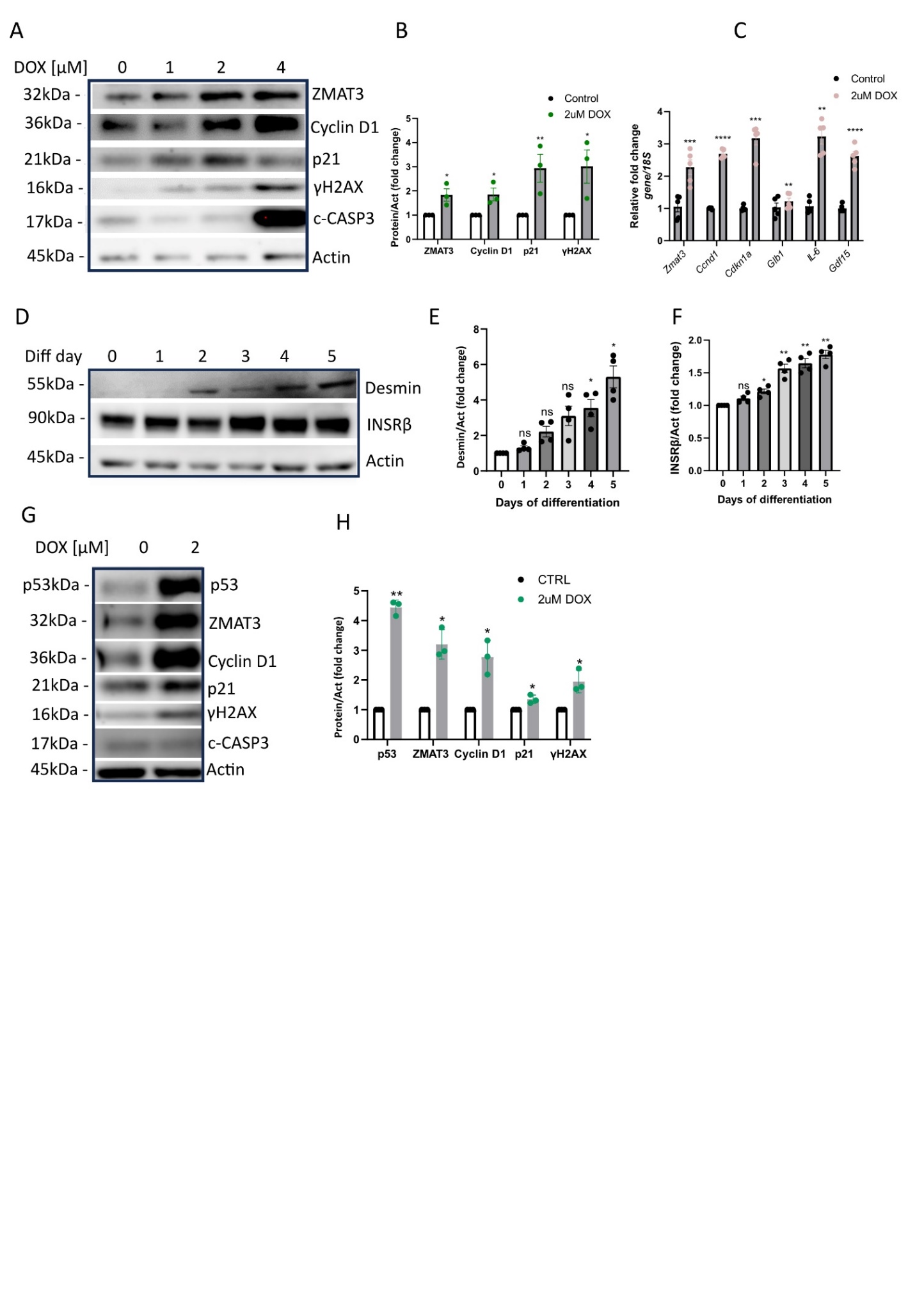


Sup. Fig. 3

(A) Representative immunoblots of the indicated proteins in control and DOX-treated C2C12 cells after 24 hours. (B) Bar graphs showing relative protein levels in control and DOX-treated cells after 24 hours, normalized to actin (n = 3). Data are presented as mean ± SEM, with dots representing individual data points. *p<0.05, **p<0.01 (Wilcoxon matched-pairs test or paired Student’s t-test). (C) Bar graphs displaying qRT-PCR analysis of the indicated genes as fold change, normalized to 18S (n = 5). Data are presented as mean ± SEM, with dots representing individual data points. **p < 0.01, ***p < 0.001, ****p < 0.0001 (Wilcoxon matched-pairs test or paired Student’s t-test). (D) Representative immunoblots of the indicated proteins upon differentiation. (E-F) Bar graphs showing relative protein levels upon differentiation, normalized to actin (n = 4). Data are presented as mean ± SEM, with dots representing individual data points. *p<0.05, **p<0.01 (Wilcoxon matched-pairs test or paired Student’s t-test). (G) Representative immunoblots of the indicated proteins in control and DOX-treated differentiated C2C12 cells. (H) Bar graphs showing relative protein levels in control and DOX-treated cells normalized to actin (n = 3). Data are presented as mean ± SEM, with dots representing individual data points. *p<0.05, **p<0.01 (Wilcoxon matched-pairs test or paired Student’s t-test).


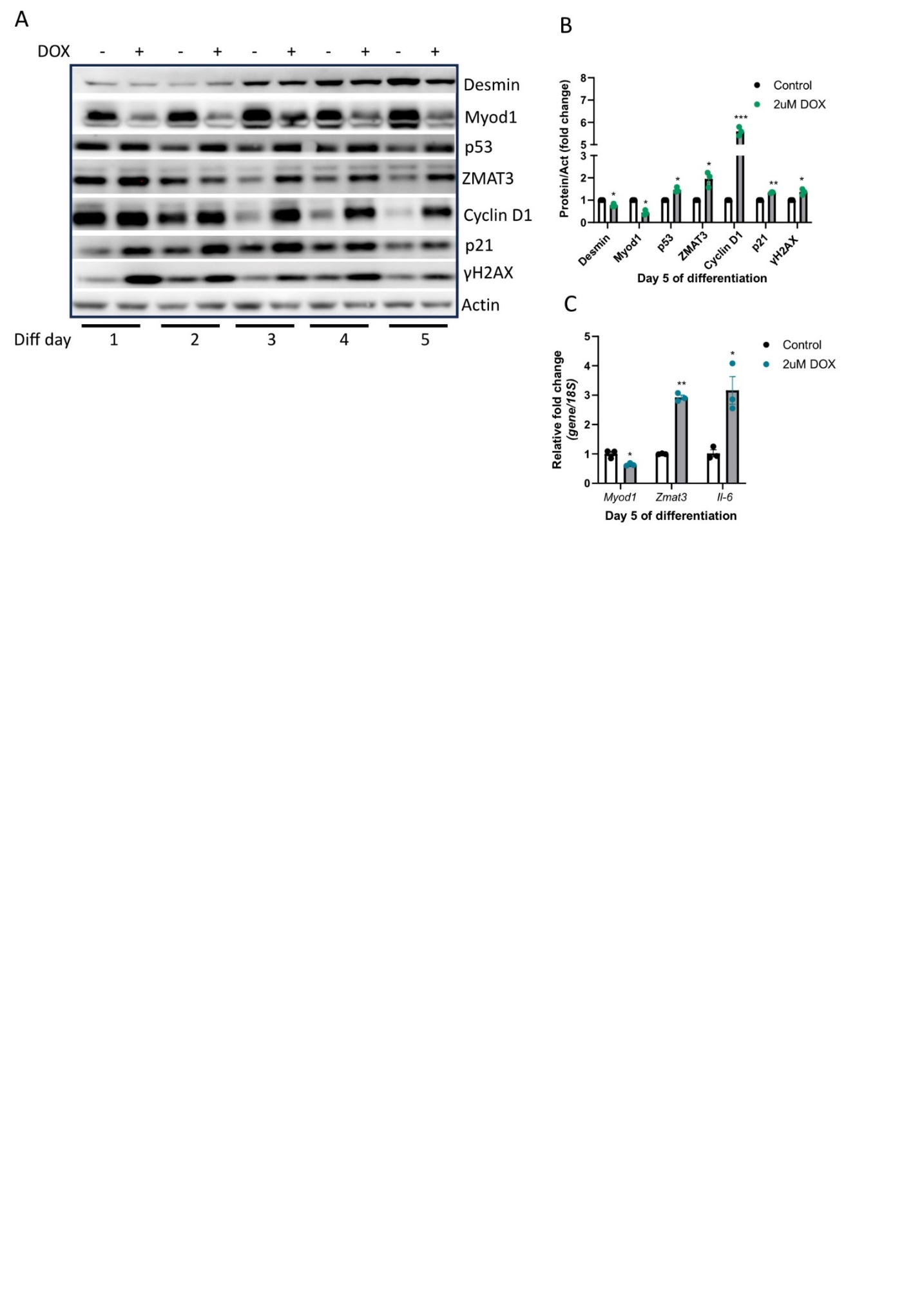


Sup. Fig. 4

(A) Representative immunoblots of the indicated proteins in control and DOX-treated C2C12 cells upon differentiation. (B) Bar graphs showing relative protein levels in control and DOX-treated cells, normalized to actin (n = 3). Data are presented as mean ± SEM, with dots representing individual data points. *p<0.05, **p<0.01, ***p < 0.001 (Wilcoxon matched-pairs test or paired Student’s t-test). (C) Bar graphs displaying qRT-PCR analysis of the indicated genes as fold change, normalized to 18S (n = 3). Data are presented as mean ± SEM, with dots representing individual data points. *p<0.05, **p < 0.01 (Wilcoxon matched-pairs test or paired Student’s t-test).
